# Annexin A7 is required for ESCRT III-mediated plasma membrane repair

**DOI:** 10.1038/s41598-019-43143-4

**Published:** 2019-04-30

**Authors:** Stine Lauritzen Sønder, Theresa Louise Boye, Regine Tölle, Jörn Dengjel, Kenji Maeda, Marja Jäättelä, Adam Cohen Simonsen, Jyoti K. Jaiswal, Jesper Nylandsted

**Affiliations:** 1 Unit for Cell Death and Metabolism, Center for Autophagy, Recycling and Disease, Danish Cancer Society Research Center, Strandboulevarden 49, DK-2100 Copenhagen, Denmark; 2 Department of Dermatology, Medical Center, University of Freiburg, 79104 Freiburg, Germany; 3 Department of Biology, University of Fribourg Chemin du Musée 10, 1700 Fribourg, Switzerland; 4 Department of Cellular and Molecular Medicine, Faculty of Health Sciences, University of Copenhagen, DK-2200 Copenhagen N, Denmark; 5 Department of Physics, Chemistry and Pharmacy, University of Southern Denmark, Campusvej 55, DK-5230 Odense M, Denmark; 6 Children’s National Health System, Center for Genetic Medicine Research, George Washington University School of Medicine and Health Sciences, 111 Michigan Avenue, NW, Washington, DC 20010-2970 USA; 7 Department of Genomics and Precision Medicine, George Washington University School of Medicine and Health Sciences, 111 Michigan Avenue, NW, Washington, DC 20010-2970 USA

**Keywords:** Breast cancer, Membrane structure and assembly, ESCRT

## Abstract

The plasma membrane of eukaryotic cells forms the essential barrier to the extracellular environment, and thus plasma membrane disruptions pose a fatal threat to cells. Here, using invasive breast cancer cells we show that the Ca^2+^ - and phospholipid-binding protein annexin A7 is part of the plasma membrane repair response by enabling assembly of the endosomal sorting complex required for transport (ESCRT) III. Following injury to the plasma membrane and Ca^2+^ flux into the cytoplasm, annexin A7 forms a complex with apoptosis linked gene-2 (ALG-2) to facilitate proper recruitment and binding of ALG-2 and ALG-2-interacting protein X (ALIX) to the damaged membrane. ALG-2 and ALIX assemble the ESCRT III complex, which helps excise and shed the damaged portion of the plasma membrane during wound healing. Our results reveal a novel function of annexin A7 – enabling plasma membrane repair by regulating ESCRT III-mediated shedding of injured plasma membrane.

## Introduction

The plasma membrane is composed of a single phospholipid bilayer, which constitutes the boundary between the eukaryotic cell and its environment. Unlike prokaryotic cells, which are protected by a cell wall, most eukaryotic cells lack a cell wall and are thus more vulnerable to membrane disruptions^1^. Hence, effective plasma membrane repair mechanisms appear to have evolved to cope with membrane lesions and ensure cell survival^2,3^. The eukaryotic repair response utilizes cytoskeletal and endomembrane systems, and involves both membrane fusion, and membrane replacement mechanisms^4–7^. Influx of Ca^2+^ ions plays a critical role as the initial trigger of repair by activating and guiding repair proteins containing Ca^2+^-binding motifs to the site of injury. Repair of both small (<100 nm) and large (micron scale) plasma membrane wounds involve shedding of damaged membrane (ectocytosis), which is facilitated by the endosomal sorting complex required for transport (ESCRT) III^8,9^.

Members of the annexin (ANXA) protein family are Ca^2+^-sensitive proteins that regulate various endomembrane processes including vesicle fusion, segregation, and compartmentalization and are implicated in plasma membrane repair mechanisms^5,10^. Annexins share the ability to fuse membranes and aggregate vesicles, and this function is important during plasma membrane repair to reseal membrane lesions. However, the role of individual annexin family members in the plasma membrane repair system is, as yet, not well characterized.

Annexins are phospholipid-binding proteins composed of a conserved COOH-terminal core domain containing four structural repeats, on which type-2 Ca^2+^-binding sites are located^11^. Upon Ca^2+^-binding, annexins undergo a conformational change allowing them to bind anionic phospholipids in membranes and interact with specific effector proteins such as EF-hand S100 proteins via their N-terminal region^12^. S100 and annexin proteins, are overexpressed in various cancers and are associated with poor overall survival, suggesting an enhanced need for membrane trafficking and repair in cancer cells^13,14^.

Annexin A7 (ANXA7), originally named synexin, was the first annexin to be discovered four decades ago^15^ and has since been associated with tumor progression and reduced overall survival in cancers including hepatocellular carcinoma and breast cancer^16,17^. However, ANXA7 also acts as a tumor suppressor gene in certain tumors such as glioblastoma multiforme^18^, melanoma^19^ and prostate cancer^20^. ANXA7 protein was originally identified to promote Ca^2+^-dependent aggregation of isolated chromaffin granules or lipid vesicles^15,21^, and has been implicated in Ca^2+^-dependent membrane fusion during exocytosis as observed in lung alveolar surfactant secretion^22,23^. Moreover, ANXA7 is associated with Ca^2+^ homeostasis, cardiac remodeling^24^, and inflammatory myopathies^25^. Compared to other annexins, ANXA7 contains an extraordinary long hydrophobic N-terminus of more than 100 amino acids^11^, which appears to confer functional specificity and is responsible for interaction with the Ca^2+^-binding EF-hand protein sorcin^26^.

Here we have identified ANXA7 as a novel plasma membrane repair regulator and examined the behavior of ANXA7 upon plasma membrane injury and reconstituted membrane interactions *in vitro* with purified proteins on artificial lipid bilayers. We found that upon plasma membrane damage triggered by digitonin, nine annexin family members translocate to the damaged plasma membrane of breast cancer cells. We show that both ANXA5 and ANXA7 are required for repair in breast cancer cells in addition to our previous results implicating ANXA4, ANXA6 and ANXA2^13^, indicating that a network of annexins are participating in the plasma membrane repair response. In the light of our previous findings, showing specific roles of individual annexin family members in repair including induction of membrane curvature triggered by ANXA4 and ANXA6^27^, and regulation of actin accumulation by ANXA2/S100A11^13^ we hypothesized that ANXA7 plays a distinct function in plasma membrane repair. Here we provide evidence that ANXA7 upon plasma membrane injury is recruited to the site of injury where it forms a complex with the Ca^2+^-binding protein apoptosis-linked gene-2 (ALG-2). We demonstrate that ANXA7 is required to recruit ALG-2 and ALG-2-interacting protein X (ALIX) to the damaged membrane. Our results show that by enabling Ca^2+^-triggered ALG-2 and ALIX recruitment at the injured plasma membrane, ANXA7 initiates the process of ESCRT III buildup at the site of injury, which is needed to shed damaged membrane during the repair process.

## Results

### Annexin family members are differentially recruited to the plasma membrane upon injury

**Figure 1:**
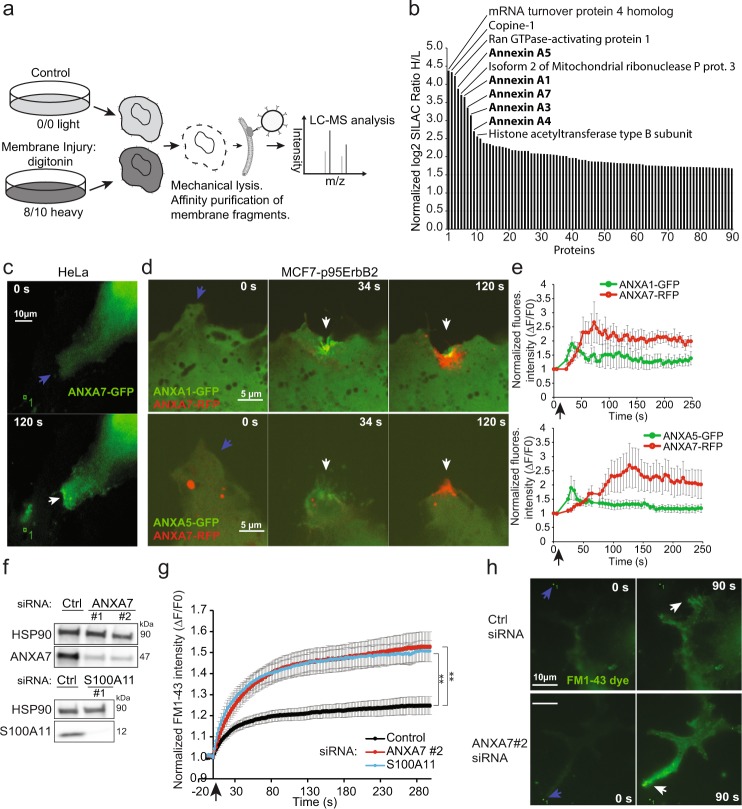
ANXA7 is needed for repair in MCF7-p95ErbB2 cells. (**a**) Schematic representation of the proteomic setup. MCF7-p95ErbB2 cells were cultured in SILAC medium. Cells were left uninjured (control) or injured by treating with digitonin (20 µg/ml) for 10 min at 37 °C. Cells were disrupted and biotin labelled membrane fragments were affinity purified using streptavidin beads before MS analysis. (**b**) Plot showing change in cell surface level of proteins presented as the ratio of MCF7-p95ErbB2 cells treated with digitonin over uninjured MCF7-p95ErbB2 as measured by MS analysis (Also see Supplementary Fig. S1A and Table 1). (**c**) Representative sequential images of HeLa cells showing the translocation of ANXA7-GFP or (**d**) ANXA7-RFP and ANXA1-GFP (upper panel) or ANXA7-RFP and ANXA5-GFP (lower panel) in MCF7-p95ErbB2 cells in response to focal laser injury (blue arrow indicates injury site). (**e**) Corresponding translocation kinetic plots. Error bars represent SEM from three independent experiments. (**f**) Immunoblot showing ANXA7 and S100A11 protein levels in MCF7-p95ErbB2 depleted for ANXA7 or S100A11, as compared to Ctrl siRNA. HSP90 served as loading control (72 h). (**g**) Plasma membrane repair kinetics upon laser injury measured by membrane impermeable FM1-43 dye influx in MCF7-p95ErbB2 transfected with ANXA7, S100A11 or Ctrl siRNA for 72 h. Error bars represent SEM for at least 10 independent cells per condition. (**h**) Representative images of MCF7-p95ErbB2 cell transfected with either Ctrl siRNA (upper panel) or ANXA7 siRNA (lower panel) showing the influx of impermeable FM1-43 dye after laser injury (white arrow indicates injury site). The asterisk represent P-values based on Student’s t-test (g): **P ≤ 0.01, as indicated when comparing indicated siRNAs to control siRNA. Full-length blots are presented in Supplementary Fig. S2b.

To identify injury-induced changes in the plasma membrane proteome we used MCF7 breast cancer cells expressing a N-terminally truncated 95 kDa version of ErbB2 (p95ErbB2), which increases their membrane dynamics and invasiveness^28,29^. MCF7-p95ErbB2 cells were metabolically labeled by stable isotope-labeling by amino acids in cell culture (SILAC) and the plasma membrane proteins were tagged by biotin. The cells were then injured in the presence of Ca^2+^, by exposing them to the membrane pore-forming detergent digitonin, and allowed to repair for 5 min at 37 °C (Fig. 1a). Plasma membrane fragments and associated proteins were purified by biotin-streptavidin affinity purification and compared to non-injured conditions by quantitative mass spectrometry analysis^8,30^. By the digitonin-injury approach we identified six known Ca^2+^-binding proteins amongst the list of ten proteins that increased most at the injured plasma membrane including: Copine-1 (CPNE1), ANXA1, ANXA3-A5, and ANXA7 (Fig. 1b). Other proteins detected included mRNA turnover protein 4 homolog (MRT4), Ran GTPase-activating protein 1, Isoform 2 of Mitochondrial ribonuclease P and Histone acetyltransferase type B subunit. However, these proteins showed high ratio variability in two independent experiments and thus were not considered further (Fig. 1b and Supplementary Table S1). Notably, amongst the list of all increased proteins at the plasma membrane upon digitonin-triggered injury, nine belonged to the annexin family including: ANXA1-A7, ANXA9, and ANXA11. Of these, ANXA4, ANXA5 and ANXA7 showed the highest fold increase in two independent experiments (Supplementary Fig. S1a). While distinct roles of ANXA4 and ANXA5 in plasma membrane repair has been demonstrated previously^27,31^, the function of ANXA7 is, yet, not characterized. Considering the localization of annexins to plasma membrane upon injury and extracellular vesicles^32,33^ we examined the involvement of ANXA7 in plasma membrane repair.

### ANXA7 depletion compromises repair of MCF7-p95ErbB2 cells

To monitor the behavior of ANXA7 in response to plasma membrane injury, HeLa cells expressing ANXA7-GFP or MCF7-p95ErbB2 co-expressing ANXA7-RFP and ANXA1-GFP/or ANXA5-GFP were exposed to local membrane injury by focal laser. ANXA7-GFP started accumulating at the repair site within 20 s, reaching a maximum accumulation by 80–120 s, whereas ANXA1-GFP and ANXA5-GFP peaked already after 30 s in MCF7-p95ErbB2 cells (Figs 1c–e and S1b). Thus, ANXA7 shows slower translocation velocity to the site of injury as compared to ANXA1 and ANXA5. Interestingly, ANXA1 appeared to enhance the translocation speed of ANXA7 in these experiments (Figs 1d,e and S1b). We then investigated whether ANXA7 is needed for repair by monitoring the kinetics of entry of the membrane-impermeable FM1-43 dye into MCF7-p95ErbB2 cells following the above focal laser injury approach. Use of ANXA7-specific siRNA allowed >90% knockdown of ANXA7, which compromised plasma membrane repair, as compared to control siRNA transfected cells (Fig. 1f–h). The compromise in repair kinetics following ANXA7 depletion was similar to that of cells depleted for S100A11 – a protein which we have previously revealed is required for the repair of MCF7-p95ErbB2 cells^13^ (Fig. 1g). In a similar assay we measured repair kinetic in cells depleted for either ANXA5 or ANXA7, or in cells depleted for both ANXA7 and ANXA5 by siRNAs. While ANXA5 or ANXA7 depletion compromised repair, ANXA5/ANXA7 dual depletion impaired repair slightly more (Supplementary Fig. S1c,d). In a complementary plasma membrane repair assay, MCF7-p95ErbB2 cells were injured by membrane pore-forming detergent - digitonin and assayed for membrane repair by monitoring the cell’s ability to exclude membrane-impermeable dye - propidium iodide. MCF7-p95ErbB2 cells depleted for ANXA7 or ANXA5 were poorer at repairing their permeabilized plasma membrane, further supporting the requirement of ANXA7 and ANXA5 for maintaining plasma membrane integrity in MCF7-p95ErbB2 cells (Supplementary Fig. S1e).

### ANXA7 is required to position ALG-2 at the site of repair

**Figure 2:**
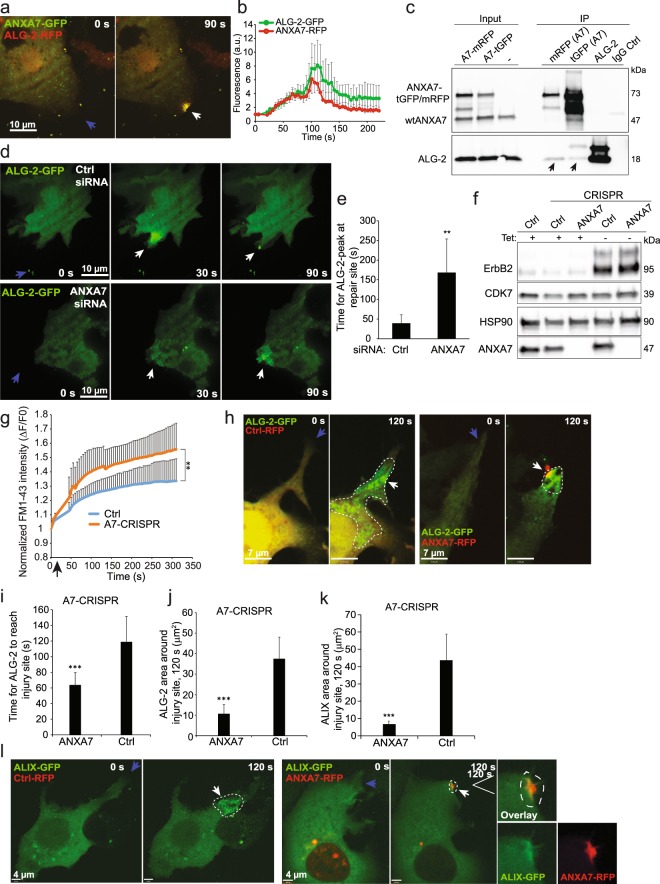
ANXA7 is needed for positioning ALG-2 at the repair site. (**a**) Representative sequential images of MCF7-p95ErbB2 cell expressing ALG-2-RFP and ANXA7-GFP exposed to laser injury (blue arrow indicates injury site) and (**b**) corresponding kinetic blot from three independent experiments. Error bars represent SEM. Also, see Supplementary Movie 1. (**c**) Representative immunoprecipitation experiment using RFP, GFP, ALG-2 or control IgG antibodies from lysates of MCF7-p95ErbB2 cells overexpressing ANXA7-mRFP or ANXA7-tGFP and exposed to scrape injury. Following IP, immunoblot analysis was carried out using ANXA7 and ALG-2 antibodies. (**d**) Representative sequential images of the translocation of ALG-2-GFP in response to laser injury (blue arrow indicates injury site) in MCF7-p95ErbB2 cells transfected with control siRNA (upper panel) or ANXA7 siRNA (lower panel) (72 h). (**e**) Time for peak of ALG-2 accumulation at the repair site following laser injury of MCF7-p95ErbB2 transfected with indicated siRNAs (72 h). Error bars represent SD for 10 independent cells per condition. The asterisk represent P-values based on Student’s t-test: **P ≤ 0.01. (**f**) Immunoblot showing ANXA7 and p95ErbB2 protein levels in MCF7-p95ErbB2 Ctrl-CRISPR cells as compared to A7-CRISPR cells. Tet– refers to cells washed to remove tetracycline to induce p95ErbB2 expression. HSP90 and CDK7 served as controls for equal loading. (**g**) Cell membrane repair kinetics upon laser injury measured by membrane impermeable FM1-43 dye influx in MCF7-p95ErbB2 Ctrl-CRISPR cells as compared to A7-CRISPR cells (A7-CRISPR cells show compromised repair). Error bars represent SD for at least 7 independent cells per condition. (**h**) Representative images of translocation behavior of ALG-2-GFP upon focal laser injury (blue arrow indicates injury site) in MCF7-p95ErbB2 A7-CRISPR cells expressing Ctrl-RFP plasmid (left panel) or wildtype ANXA7-RFP (right panel). (**i**) Time for ALG-2 to reach injury site in MCF7-p95ErbB2 A7-CRISPR cells expressing Ctrl-RFP plasmid or wildtype ANXA7-RFP. (**j**) ALG-2-GFP distribution (µm^2^) or (**k**) ALIX-GFP distribution (µm^2^) around the injured membrane in A7-CRISPR cells expressing either Ctrl-RFP or ANXA7-RFP measured by Volocity software. (**l**) Representative sequential images of ALIX-GFP translocation upon laser injury (blue arrow indicates injury site) in MCF7-p95ErbB2 A7-CRISPR cells expressing Ctrl-RFP plasmid (left panel) or wildtype ANXA7-FP (right panel). Note, ANXA7-RFP tend to form unspecific aggregates when overexpressed (red puncta around the nucleus). The aggregates are inert and do not respond to membrane injury. White line indicates region of interest (ROI). Error bars represent SD for at least 5–6 independent experiments. P-values based on Student’s t-test: ***P ≤ 0.001. Full-length blots for 2c and 2f are presented in Supplementary Figs S2c and S3 respectively.

To gain insight into the mechanism by which ANXA7 regulates repair, and in the light of the potential interaction between the ESCRT III regulator, ALG-2 and the N-terminal end of ANXA7^34^ we monitored ANXA7-GFP and ALG-2-RFP translocation upon laser injury in MCF7-p95ErbB2 cells. Interestingly, ANXA7-GFP accumulated at the damaged membrane and co-localized with ALG-2-RFP at the site of injury and similar translocation pattern was observed for ANXA7-RFP/ALG-2-GFP (Fig. 2a,b and Supplementary Fig. S2a and Movie S1). To test interaction between ANXA7 and ALG-2 proteins, we carried out co-immunoprecipitation (co-IP) experiments in MCF7-p95ErbB2 cells overexpressing ANXA7-RFP or ANXA7-GFP using anti- mRFP or tGFP antibodies (Figs 2c and S2c). Following scrape injury, ANXA7 was able to co-IP endogenous ALG-2 using both, tGFP and mRFP antibodies (Fig. 2c), whereas siRNA-depletion of ALG-2 reduced ANXA7 co-immunoprecipitation of ALG-2 (Supplementary Fig. S2e). Thus, these results suggest that ANXA7 and ALG-2 can form a complex in injured cells.

To examine if ANXA7 is required for injury-triggered trafficking of ALG-2 at the site of injury, we monitored the ALG-2-GFP response to injury following siRNA-mediated depletion of ANXA7 in MCF7-p95ErbB2 cells. Lack of ANXA7 (<10%) delayed the translocation of ALG-2-GFP to the site of injury, and unlike the control cells where ALG-2 cleared out from the injury site, in ANXA7 depleted cells ALG-2 remained accumulated in a spatially diffuse manner around the injured membrane (Fig. 2d,e). To complement this further we generated MCF7-p95ErbB2 cells with CRISPR/cas9 disrupted ANXA7 gene expression (A7-CRISPR) (Fig. 2f,g) and attempted to rescue the ANXA7 knockout phenotype by re-expressing ANXA7-RFP or Ctrl-RFP. ANXA7-RFP reversed the slower translocation of ALG-2-GFP to the injury site (Figs 2h,i and S2d), and caused localized accumulation of ALG-2-GFP at the injury site (Fig. 2h, right panel and Fig. 2j). In contrast, expression of Ctrl-RFP resulted in a diffuse and more widespread distribution of ALG-2-GFP in cells indicating that its localization at the damaged membrane depends on ANXA7 (Fig. 2h, left panel and Fig. 2j). Since ALG-2 mediates injury-triggered ALIX accumulation^8^, we next examined if ANXA7 knockout also alters ALIX accumulation at the site of injury. Simultaneous imaging of ALIX-GFP and ANXA7-RFP or Ctrl-RFP in A7-CRISPR cells showed that in the Ctrl-RFP expressing cells, ALIX-GFP accumulated in a spatially diffuse manner (similar to ALG-2), whereas expression of ANXA7-RFP caused co-localized accumulation of ALIX-GFP at the site of damage (Fig. 2k,l). Thus, ANXA7 appears to be required for proper temporal and spatial translocation of ALG-2 and ALIX to the site of injury.

### ALG-2 is dependent on ANXA7 for membrane binding

**Figure 3:**
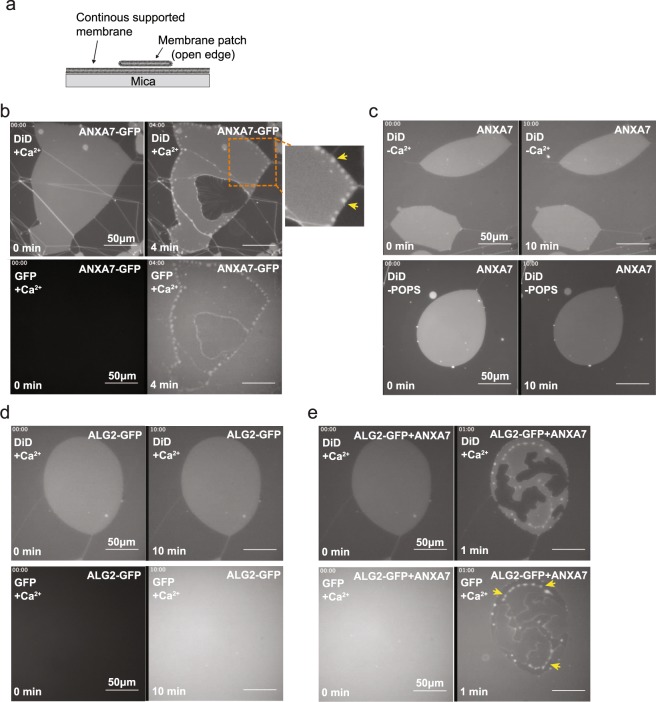
ANXA7 is required for ALG-2 binding to supported membranes. (**a**) Schematic representation of supported membrane model composed of primary and secondary membranes. Non-vesicular membrane patches with open edges were used in the subsequent experiments (POPC/POPS, 9:1 molar ratio). (**b**) Representative sequential images before and after addition of recombinant ANXA7-GFP protein to a membrane patch stained with DiD (DiD channel: upper panel. Lower panel: GFP channel) in the presence of Ca^2+^ (Also see Supplementary Movie S2). Note, ANXA7-GFP induces a pearl-like membrane conformation. (**c**) Similar experiment as in b but in the absence of Ca^2+^ (top panel) or POPS (lower panel). (**d**) Sequential images before and after addition of recombinant ALG-2-GFP protein to a membrane patch stained with DiD in the presence of Ca^2+^. (**e**) Addition of ANXA7 (without GFP) and ALG-2-GFP proteins trigger ALG-2-GFP membrane binding. Accumulation of ALG-2-GFP into pearl-like peripheral structures induced by ANXA7 are indicated by yellow arrows (upper panel, DiD. Lower panel: GFP channel).

To examine the ability of ANXA7 to recruit ALG-2 directly on membranes we attempted to reconstitute this process *in vitro*. We generated recombinant ANXA7 and ALG-2 proteins and used them together with artificial solid supported lipid membrane patches formed by hydration of the dry precursor lipid film^35^. These membrane patches contain freestanding edges allowing us to model the edge of an injured plasma membrane (Fig. 3a). Recombinant ANXA7-GFP protein added to these membranes accumulated locally (in <4 min) at the free membrane edges into a pearl-like pattern and caused these edges to curve (Fig. 3b and Supplementary Movie S2). This effect of ANXA7 required presence of Ca^2+^ and negatively charged phosphatidylserine in the membrane (Fig. 3c). Adding recombinant ALG-2-GFP protein to these freestanding membranes showed that ALG-2 failed to bind or have any effect on membrane conformation, demonstrating the inability of ALG-2 to bind the membrane on its own (Fig. 3d). However, when recombinant ALG-2-GFP and ANXA7 proteins were co-incubated, ALG-2-GFP accumulated into pearl-like structures at the free membrane edges (Fig. 3e). Taken together, these results show that ANXA7 binds to the free membrane edges in a Ca^2+^- and phosphatidylserine-dependent manner and facilitates ALG-2 binding to the membrane.

### ANXA7 is required for ESCRT III mediated shedding of damaged membrane

**Figure 4:**
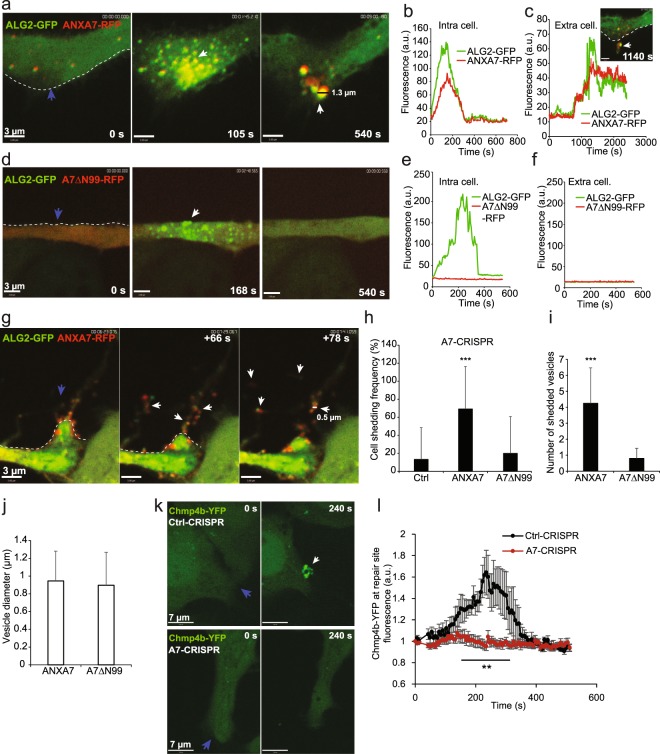
ANXA7 is needed for Chmp4b buildup and ectosome shedding. (**a**) Representative sequential images of the translocation behavior and shedding of ANXA7-RFP and ALG-2-GFP in MCF7-p95ErbB2 A7-CRISPR upon localized laser injury and corresponding intensity plots of proteins intracellularly (**b**) or extracellularly (**c**) measured by Volocity software (blue arrow indicates injury site). (**d-f**) similar experiment as in a, but with ANXA7 mutant (A7ΔN99) lacking most of the N-terminus. Note, this mutant have decreased Ca^2+^-sensitivity and shedding activity and fail to localize ALG-2-GFP at the injured membrane. (**g**) Typical sequential images showing ANXA7-RFP/ALG-2-GFP translocation to injured membrane and subsequently excision and shedding of membrane containing protrusions. (**h**) Cell shedding frequency in MCF7-p95ErbB2 A7-CRISPR cells expressing Ctrl-RFP, wildtype ANXA7-RFP or A7ΔN99-RFP mutant. Error bars represent SD for at least 15 independent cells per condition. P-values based on Student’s t-test: ***P ≤ 0.001. (**i**) Number of shedded vesicles as measured from the very edge of the cells membrane upon laser injury of MCF7-p95ErbB2 A7-CRISPR cells expressing wildtype ANXA7-RFP or A7ΔN99-RFP mutant. Cells were injured at the very edge of the cell membrane and shedded vesicles captured by time lapse imaging (Also see Supplementary Movies S3–S5). (**j**) Average vesicle diameter of shedded vesicles after laser injury. Examples of vesicle diameter indicated in a and c. Data were obtained from time-lapse movies using Volocity software. SD for at least 4 experiments. (**k**) Typical sequential images of Chmp4b-YFP translocation in MCF7-p95ErbB2 Ctrl-CRISPR or A7-CRISPR cell upon laser injury. (**l**) Graph showing Chmp4b-YFP translocation kinetic at the site of injury in MCF7-p95ErbB2 Ctrl-CRISPR and A7-CRISPR cells. Error bars represent SEM for at least 8 independent cells per condition. P-values based on Student’s t-test: **P ≤ 0.01.

While we find that the artificial membrane edges bind ANXA7/ALG-2 complex, we have previously identified that ALG-2 enables membrane shedding *in vivo* by facilitating accumulation of ALIX and ESCRT III protein complex at the injured plasma membrane^8^. To elucidate this in cells we tested if an ANXA7 mutant (ANXA7ΔN99) lacking 99 amino acids of the N-terminal region responsible for ALG-2 binding while maintaining the core annexin domains intact, can facilitate injury-triggered ALG-2 accumulation at the injury site^34,36^. MCF7-p95ErbB2 A7-CRISPR cells were transfected with ALG-2-GFP and either full length ANXA7-RFP or ANXA7ΔN99-RFP. Full-length ANXA7 and ALG-2 complex accumulated at the injury site and was subsequently shed with damaged membrane as the cell repaired (Fig. 4a–c). In contrast, ANXA7ΔN99-RFP mutant failed to translocate to the injured membrane, which prevented translocation of ALG-2 to the site of injury (Fig. 4d-f). This demonstrates that the N-terminus of ANXA7 is important for translocation to the damaged membrane and Ca^2+^-mediated membrane binding of ALG-2, as suggested previously^36^. In response to ANXA7/ALG-2 binding, vesicles were typically shed within 2–15 min (Fig. 4g, Supplementary Movies S3–S5) as monitored by confocal imaging in accordance with previous studies^8,9^. Next, we monitored the shedding frequency upon laser injury. While 70% of cells with wildtype ANXA7-RFP shed their injured plasma membrane, this only occurred in 15–20% of Ctrl-RFP or ANXA7ΔN99-RFP expressing cells (Fig. 4h). Moreover, the number of shedded vesicles were significantly reduced in the ANXA7ΔN99-RFP mutant as compared to wildtype ANXA7-RFP, whereas the size of shedded vesicles were similar in the two conditions (Fig. 4i,j). Since ALG-2 enables ALIX-mediated recruitment of ESCRT III protein complex at the injured plasma membrane^8^, we used live cell imaging to monitor the behavior of Chmp4b – a core component of the ESCRT III complex. Upon injury, MCF7-p95ErbB2 Ctrl-CRISPR cells accumulated Chmp4b-YFP puncta at the damaged membrane within 240 s, whereas MCF7-p95ErbB2 A7-CRISPR cells were impaired in their ability to localize Chmp4b at the repair site (Fig. 4k,l). These results suggest that ANXA7 is responsible for binding and positioning ALG-2 and ALIX to the injured membrane and this function is important for assembly of ESCRT III components and subsequent shedding of damaged membrane.

## Discussion

Our proteomic analysis of invasive breast cancer cells shows that annexin family members are amongst the proteins that show greatest enrichment at the plasma membrane during repair. Differences in the enrichment of annexins at the plasma membrane may be due to variances in their Ca^2+^-sensitivity^37^ and their binding affinity to the plasma membrane. Annexin family members have been associated with plasma membrane repair, but their precise mechanisms of action are poorly characterized^5^. It seems plausible that several annexins act in concert to repair the damaged membrane, which our data support. However, besides their shared ability to aggregate and fuse membranes, which is important for repair, annexins appear to serve more specific functions in plasma membrane repair^5^. Previously we have shown that annexin family member, ANXA2 together with its binding partner S100A11, stabilizes the injured plasma membrane by facilitating F-actin buildup at the wound site^13^. This role of ANXA2 in MCF7-p95ErbB2 cells is distinct from our more recent finding of a biophysical role of ANXA4^27^, another annexin family member that we find is highly enriched at the repairing plasma membrane. ANXA4 is recruited to the damaged membrane and homo-trimerizes at the wound edges, inducing membrane curvature. This curved membrane then undergoes constriction by ANXA6, which helps pull the wounded membrane edges together for eventual closure^27^. Here, we add further to the specialized annexin functions during the repair process, which helps cells to cope with membrane disruptions. We show that a novel function of annexin family member ANXA7 is to facilitate ESCRT III-mediated shedding of the damaged edges of the wounded plasma membrane, enabling membrane repair. ANXA7 facilitates this by regulating the binding of ALG-2 to the injured plasma membrane. ALG-2 in turn enables recruitment of ALIX, which then directly binds and recruits the ESCRT III proteins^8^. Thus, ANXA7 appears to be part of the early repair response, regulating proper localization of ALG-2 and ALIX at the damaged membrane. This is supported by *in vitro* reconstitution of ANXA7 facilitated recruitment of ALG-2 in the tethered membrane patch approach. ANXA7 and ALG-2 accumulate locally at the edge of the membrane patch, causing formation of a pearl-like pattern of the bound membrane triggered by ANXA7. This ability is unique for ANXA7 and ANXA11 as compared to other annexin family members and involve induction of local membrane curvature as measured by atomic force microscopy^35^. The pearl/lens structures are fluid, mobile, and predominantly nucleated near the membrane edges where they appear to co-localize with phosphatidylserine lipid between the two membrane leaflets in the bilayer. The occasional rupture of lenses and release of their content suggests that they encapsulate a hydrophobic complex of phosphatidylserine and annexin such as reverse micelles or a related geometry^35^. Formation of big fluid lenses is only associated with the tethered membrane patch model and, hence is not observed in cells. However, in cells ANXA7 forms local pearl-like structures together with ALG-2 at the damaged membrane before buildup of ESCRT III complex. Since membrane bending is canonical for ESCRT III-mediated abscission^38^, ANXA7 might help in this process by inducing local membrane curvature needed to initiate ESCRT III complex-assembly and subsequently local shedding of membrane in the form of ectosomes. However, the exact nature of this mechanism remains to be fully elucidated.

**Figure 5:**
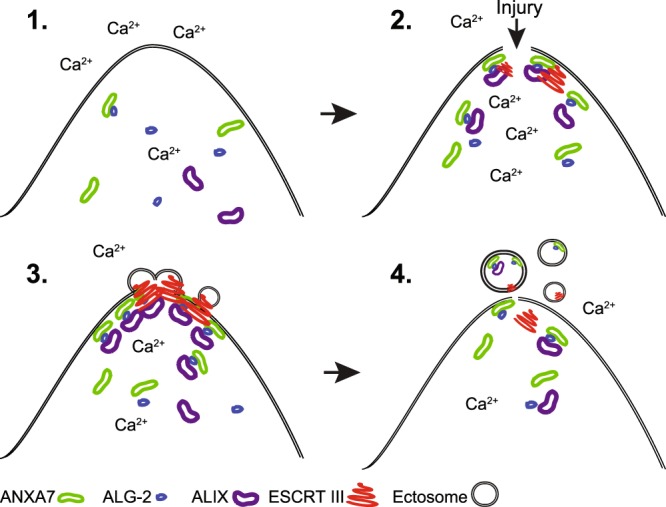
Proposed model for plasma membrane repair mediated by ANXA7. 1. In resting cells ANXA7, ALG-2 and ALIX are predominantly cytosolic. 2. Following plasma membrane injury and influx of Ca^2+^ into the cytoplasm ANXA7, ALG-2 and ALIX forms a complex, which is anchored to the injured membrane through the core domain of ANXA7. 3. Here, ALG-2 and ALIX initiates the sequential assembly of ESCRT III components by recruiting Chmp4 proteins including Chmp4b, which assemble into membrane bound spring-shaped filaments. 4. Membrane excision occur upon contraction of the spiral structure leading to shedding of the damaged membrane. Other annexin family members including ANXA4, ANXA5 and ANXA6 participate in the repair network and are needed for wound healing (not shown here).

Based on our results we propose a model that depicts the steps in the repair response (Fig. 5). In resting cells, ANXA7, ALG-2, ALIX, and ESCRT III proteins are all predominantly cytosolic. Following plasma membrane injury, influx of Ca^2+^ into the cytoplasm causes ALG-2 to bind to the N-terminal region of ANXA7 and form a complex with ALIX, which is anchored to the injured membrane through the phospholipid-binding core domain of ANXA7. Here, ALG-2/ALIX initiate the assembly of ESCRT III components by recruiting ESCRT III members such as Chmp4b. The ESCRT III proteins may then assemble spirally around the curved membrane followed by contraction, which lead to the shedding of the damaged membrane edges in way of ectosomes (Fig. 5). With the ubiquity of ANXA7 expression and plasma membrane injury, the role of ANXA7 in mediating plasma membrane repair by recruiting ESCRT III machinery may be broadly relevant, but heightened further in invasive breast cancer cells. Thus, our work here identifies a novel role of ANXA7 in cell survival, which may open for novel approaches to target aggressive breast cancer cells by inhibiting plasma membrane repair.

## Methods

### Cell culture and treatments

HeLa cells originating from cervix carcinoma (ATCC) were cultured in RPMI 1640 Glutamax^TM^ medium (Gibco) supplemented with 6% heat-inactivated fetal calf serum (Biological Industries), penicillin and streptomycin. MCF7-p95ErbB2 cell line is single cell clone of MCF7 cells stably expressing the tetracycline transactivator with a truncated version of ErbB2 (p95ErbB2) in the pTRE plasmid^39^. p95ErbB2 expression is induced by washing off tetracyclin (1 µg/mL) with PBS. All cells were kept at 37 °C in a humidified atmosphere of 5% CO2. The experiments were carried out at 3–5 passage after the induction and in all the experiments with p95ErbB2 refers to induced cells.

### siRNAs and plasmid transfection

siRNA transfections were performed using oligofectamine^TM^ transfection reagent (ThermoFischer) with 25 nM siRNA (Sigma-Aldrich), according to the manufacturer’s protocol. siRNA sequences: Control siRNA (AllStar Negative Control siRNA, Qiagen), ANXA5 (ANXA5#1: 5′-GCA ACU ACU CCU UGC UGUU[dT][dT]-3′, ANXA5#2: 5′-GUG AGA UUG AUC UGU UUAA[dT][dT]-3′), ANXA7 (ANXA7#1: 5′-CUU CAU GCC UCC UAC GUAU[dT][dT]-3′, ANXA7#2:5′-GCU UAU UCU AGG AUG GCUA[dT][dT]-3′), S100A11#1 (5′-ACU CUC UCC AAG ACA GAG UUC CUAA-3′), ALG-2 (5′-CAC GAC AUC CUC AUU CGAA[dT][dT]-3′). For plasmid transfection, cells were transiently transfected with the indicated plasmid using Lipofectamine^TM^ LTX transfection reagent (ThermoFischer) 24 h before experimentation, according to the manufacturer’s protocol.

### Immunoblotting, immunoprecipitation and antibodies

Cells were extracted in SDS-lysis buffer and proteins separated by SDS–PAGE using precast 4–15% gradient gels (BioRad) and blotted onto nitrocellulose membranes (BioRad) using Trans-Blot Turbo^TM^ transfer system and blocked in 5% milk. Primary antibodies raised against human ANXA7 (1:1000 dilution, ab49838, Abcam), ALG-2 (PDCD6, 1:1000 dilution, ab133326, Abcam) ANXA5 (1:1000 dilution, ab54775, Abcam or 1:1000 dilution ab14196, Abcam), S100A11 (1:1000 dilution, 10237-1-AP, Proteintech Europe), ErbB2 (HER-2/c-erbB-2/neu Ab-17, 1:1000 dilution, MS-730-P0-A, ThermoFisher Scientific), Heat-shock protein 90 kDa (Hsp90, 1:4000 dilution, 610418, BD Transduction Laboratories™), GAPDH (1:5000 dilution, ab189095 Abcam), CDK7 (MO-1 was kindly provided by Jiri Bartek), were used followed by appropriate peroxidase-conjugated secondary antibodies (DAKO). Immunoprecipitation: Lysates from MCF7-p95ErbB2 cells overexpressing ANXA7-monomericRFP or ANXA7-turboGFP were harvested in lysis buffer (50 mM Tris-HCl pH (7.4), 150 mM NaCl, 0. 5% Np40 (IGEPAL), 1 mM EDTA) supplemented with 1X protease (Roche) and phosphatase (Active Motif) inhibitors. Proteins were immunoprecipitated from whole cell lysate using 3 µg antibody (tGFP, TA150041, Origene) coupled to protein A agarose beads (Millipore) or using RFP-Trap agarose beads (Chromotek) for o/n at 4 °C according to the manufacturer’s instructions. The precipitates were washed four times and eluted with 2XLSB with 100 mM dithiothreitol (DTT).

### Plasmid constructs and recombinant proteins

Expression plasmids with turbo-GFP C-terminal tag containing human ANXA5, ANXA7 or ALG-2 cDNA were purchased from OriGene Technologies, and fluorescently tagged ALG-2 (RFP) plasmid was kindly provided from Hideki Shibata (Nagoya University) and Chmp4b-(YFP) from Martin Serrano (University College London). ANXA1-GFP was kind gift from Annette Draeger. ANXA7 with monomeric RFP C-terminal tag was made by subcloning of ANXA7 cDNA into the mammalian pCMV- AC-mRFP vector (OriGene). ANXA7 deletion mutants lacking amino acids 2–100 (ΔN99) of the N-terminal region were generated by PCR-based site-directed mutagenesis using QuikChange Lightning Site-Directed Mutagenesis Kit (Agilent Technologies) according to the manufacturer’s protocol. Primer sequences for generation of deletion mutant: ANXA7ΔN99, Forw. 5′-GCC GCG ATC GCC ATG GGC TTT TCT GGG TAT-3′; Rev. 5′-ATA CCC AGA AAA GCC CAT GGC GAT CGC GGC-3′. Recombinant proteins were produced from ANXA7 cDNA constructs subcloned into the bacterial pEX-C-His vector (OriGene). Moreover, for C-terminal GFP-tagged proteins ANXA7 and ALG-2 cDNA were subcloned into pETM11SUMO3sfGFP^40^. Proteins were produced using Immobilized Metal-Affinity Chromatography (IMAC) followed by fast protein liquid chromatography (FPLC). Targeted gene disruption of ANXA7 expression in MCF7-p95ErbB2 cells was performed by CRISPR/Cas9 system with the following target sequences: 5′-CGTCTCTATCAAGCTGGTGAGG-′3. Cells were transfected with the All-in-One CRISPR/Cas9 plasmid (SigmaAldrich) using Lipofectamine^TM^ LTX (ThermoFischer) according to the manufacturer’s protocol and cell-sorted for GFP expression to obtain single cell clones. Clones were verified for disruption of reading frame by sequencing and analyzed for protein expression.

### Quantitative SILAC mass spectrometry

For SILAC labeling: MCF7-p95ErbB2 cells were cultured for at least 6 generations in medium containing regular Lysine and Arginine isotopes (unlabelled) or with media containing 13C6 Arginine and 15N2,13C6 Lysine as the sole source of these amino acids (labelled) (Silantes Gmbh). Biotin-labeling of cell surface proteins: Cells grown in 150 mm dishes (approx. 1.5 × 10^7^ cells) were washed three times in ice cold HBSS (+Ca^2+^) (Gibco) and incubated for 30 min on ice at 4 °C on a rocking table with 5 mL of HBSS (+Ca^2+^) containing 1 mg/mL (approx. 2 mM biotin reagent) EZ-Link sulfo-NHS-LC-LC-Biotin (ThermoFisher) according to the manufacturer’s protocol. Cells were washed three times with cold HBSS (+Ca^2+^). Then cells were treated with 20 µg/mL digitonin (Sigma-Aldrich) in HBSS (+Ca^2+^) for 10 min at 37 °C. Control cells were quickly harvested in 10 mL cold HBSS (−Ca^2+^) and spun down. Control and injury treated cells were mixed and resuspended in 1 mL cold HBSS (+Ca^2+^) supplemented with protease (Roche) and phosphatase (Active Motif) inhibitors. Cells were disrupted by glass beads by vortexing for 2 min and kept on ice. Lysate were then spun down (750 × g at 4 °C for 10 min) to remove of nuclei and unbroken cells and the supernatant was isolated. The supernatant (=light membrane fraction including plasma membrane fragments) was incubated with 100 µl pre-equilibrated streptavidin agarose beads (Invitrogen) and incubated for 1 h at 4 °C with rotation followed by three washes in HBSS (+Ca^2+^) supplemented with protease inhibitors. Proteins were eluted in 2xLSB +5% DTT (Sigma-Aldrich) and boiled at 97 °C for 8 min. Samples were concentrated on a 10 kDa cutoff filter (Sartorius) and disulfide bonds were reduced with 1 mM DTT and alkylated using 5.5 mM iodoacetamide (Sigma–Aldrich) for 30 min at 25 °C. Proteins were separated by 4–12% Bis–Tris gradient SDS–PAGE gels (Invitrogen). Gel lanes were cut into 10 fractions and proteins therein were digested with trypsin^41^ (Promega). Peptide solutions were desalted on STAGE Tips as described^42^.

Mass Spectrometry: Peptides were analysed with a LTQ Orbitrap XL mass spectrometer (Thermo Fisher Scientific) essentially as described^43^. Briefly, an Agilent 1200 nanoflow–HPLC with a fused silica column packed in- house with Reprosil–Pur 120 ODS–3 (Dr. Maisch) was used for peptide separation prior to MS analysis. Data was acquired in the data–dependent mode and switched automatically between MS and MS/MS whereas a maximum of  1 × 10^6^ ions in MS was allowed. MaxQuant based identification and quantification of proteins

MaxQuant software version 1.4.1.2.^44^ was used for raw mass spectral data search for identification and quantification of peptides and proteins using default settings with minimally one unique peptide used for protein identification. Implemented in MaxQuant was a UniProt Homo Sapiens database from July 2015.

### Membrane wounding experiments

Laser injury assay: cells were injured at 37 °C in cell imaging media (CIM: Hank’s balanced salt solution +10 mM Hepes +2 mM Ca^2+^, pH 7.4) by pulsed UV-laser (Rapp OptoElectronic or visible light (532 nm)-laser (AblateTM, 3i Intelligent Imaging Innovations, Inc. Denver, CO, USA) by irradiating a small region (1–2 µm^2^) for <10–100 ms. The injury response was imaged with a 63x objective using either Nikon confocal microscope equipped with a PerkinElmer spinning disk (for Rapp OptoElectronic pulsed UV-laser) or IX81 Olympus microscope equipped with a Yokogawa spinning disk confocal system (for AblateTM pulsed UV laser). The cells were imaged every 4–10 s starting before injury and continuing up to 20 min following injury. Microscope hardware Olympus or Nikon was controlled by Slidebook 5.0 (Intelligent Imaging Innovations Inc., Denver) or Volocity (PerkinElmer), respectively. Slidebook or Volocity software was used to measure kinetic of repair by monitoring uptake of impermeable cellular FM1-43 dye (Life technologies) (1 mg/mL) as a change in fluorescence during the course of imaging. Volocity was used to analyze the translocation kinetic for ANXA7, ALG-2 and Chmp4b after injury, measure distribution of ALG-2 and ALIX after injury and measure cell shedding frequency. To measure membrane integrity upon digitonin permeabilization cells were incubated with 2.5 µg/mL Hoechst-33342 (Life Technologies) (Excitation 350, Emission 461) and 2 µg/mL propidium iodide (Sigma-Aldrich) (Excitation 535, Emission 617) and integrity was measured using Celigo cytometer (Brooks Life Science Systems) according to the manual and analyzed using Celigo Software Version 2.1.

### Supported membrane experiments

Sheets of mica substrates (5 × 5 mm^2^, Plano GmbH) were glued to glass coverslips (Ø24 mm, 0.17 mm thick) using the silicone elastomer MED-6215 (Nusil Technology). A droplet (40 µL) of the lipid stock was applied to the freshly cleaved mica, spun on a spincoater and placed under vacuum in a desiccator (10–12 hours) to ensure evaporation of the solvent. Dry spin-coated lipid films composed of phosphatidylcholine (POPC; 1-hexadecanoyl-2–(9Z-octadecenoyl)-sn-glycero-3-phosphocholine) and phosphatidylserine (POPS; 1-hexadecanoyl-2–(9Z-octadecenoyl)-sn-glycero-3-phospho-L-serine) (Avanti) (or without POPS) were prepared from a stock solution containing 10 mM total lipid (POPC, POPS, 9:1 molar ratio) and 0.5% DiD probe (Avanti). The spincoated lipid film was hydrated in 50 mM TRIS buffer (2-Amino-2-(hydroxymethyl)propane-1,3-diol), 100 mM NaCl, +/−2 mM CaCl_2_, pH = 7.4 at 55 °C for 2 hours. Then the sample was gently flushed with 55 °C buffer and buffer exchanged >10 times to prepare defined secondary bilayer patches resting on a continuous primary membrane. The response of bilayer patches to addition of recombinant ANXA7 or ALG-2 (produced as previously described) was monitored at 22 °C with time-lapse epi-fluorescence microscopy using a Nikon TE2000 inverted microscope (40X objective, Nikon ELWD, Plan Fluor, NA = 0.6) and DiD-excitation with a Xenon lamp (Polychrome V, Till Photonics) at 640 nm and recorded with an EMCCD camera (Sensicam EM, PCO) operated with Live Acquisition software (FEI, GmbH). ANXA7 and ALG-2 in an absolute amount of 100 pmole was added to the fluid cell and the sample imaged at 3–10 fps depending on the response speed. GFP-labeled ANXA7 or ALG-2 were imaged simultaneously to DiD using a dual wavelength filtercube.

## Supplementary information

Supplementary Information

Movie 1

Movie 2

Movie 3

Movie 4

Movie 5

## Data Availability

All data are available on request.
