# Supplementary material for: Annexin A7 is required for ESCRT III-mediated plasma membrane repair
Source: Sci Rep. 2019 Apr 30;9:6726. doi: 10.1038/s41598-019-43143-4 (PMC6491720; doi:10.1038/s41598-019-43143-4)
Supplement: Supplementary file 1 — Supplementary Information [file 41598_2019_43143_MOESM1_ESM.pdf]

SUPPLEMENTARY INFORMATION

Annexin A7 is required for ESCRT III-mediated plasma membrane repair

Authors:

Stine Lauritzen Sønder[1], Theresa Louise Boye[1], Regine Tölle[2, 3], Jörn Dengjel[2, 3], Kenji Maeda[1], Marja Jäättelä[1, 4], Adam Cohen Simonsen[5], Jyoti K. Jaiswal[6, 7], and Jesper Nylandsted[1, 4*].

Affiliations:

[1]Unit for Cell Death and Metabolism, Center for Autophagy, Recycling and Disease, Danish Cancer Society Research Center, Strandboulevarden 49, DK-2100 Copenhagen, Denmark.
[2]Department of Dermatology, Medical Center, University of Freiburg, 79104 Freiburg, Germany.
[3]Department of Biology, University of Fribourg Chemin du Musée 10, 1700 Fribourg, Switzerland.
[4]Department of Cellular and Molecular Medicine, Faculty of Health Sciences, University of Copenhagen, DK-2200 Copenhagen N, Denmark.
[5]Department of Physics, Chemistry and Pharmacy, University of Southern Denmark, Campusvej 55, DK-5230 Odense M. Denmark.
[6]Children's National Health System, Center for Genetic Medicine Research, and
[7]Department of Genomics and Precision Medicine, George Washington University School of Medicine and Health Sciences, 111 Michigan Avenue, NW, Washington, DC 20010-2970, USA.

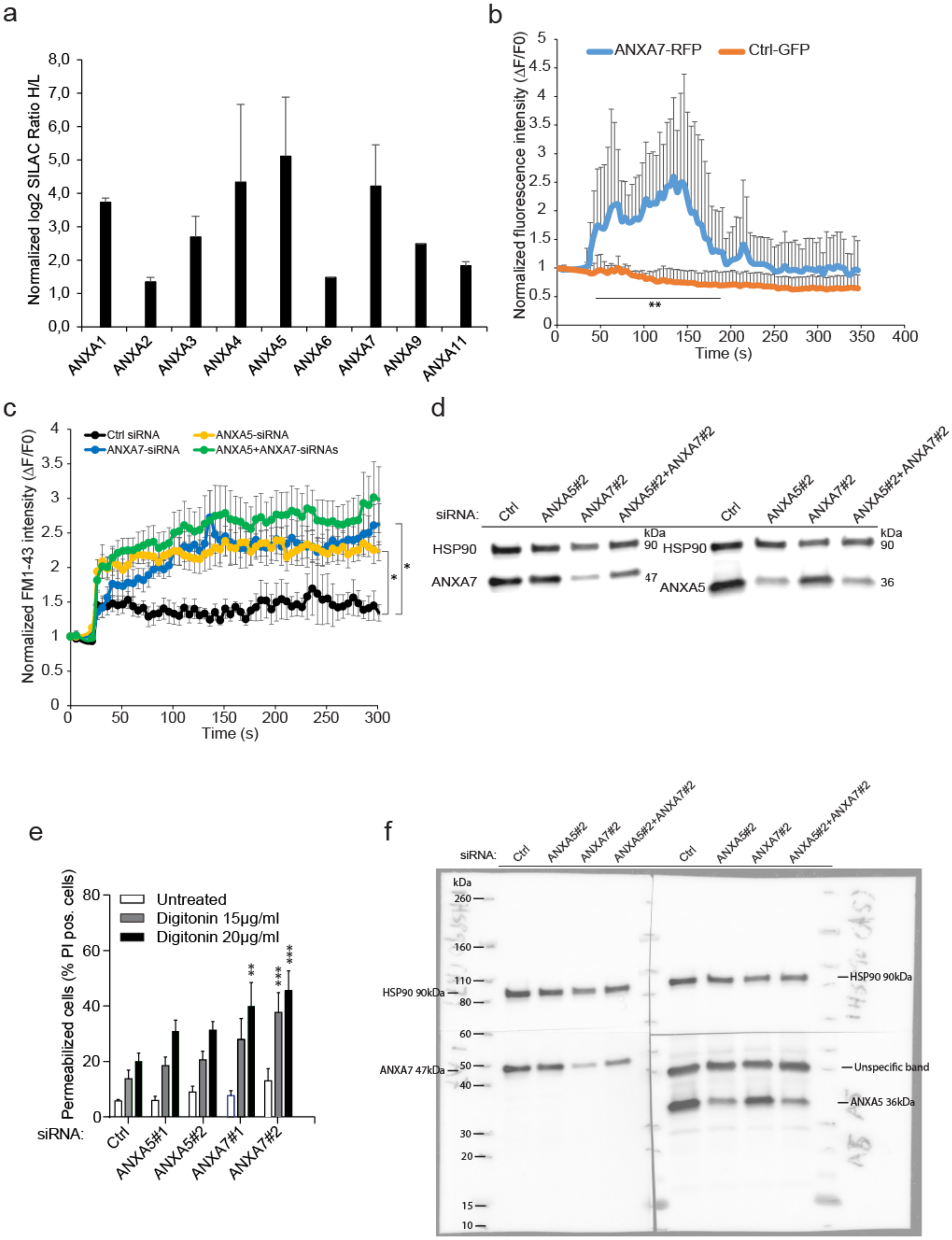

Supplementary Figure 1 (**a**) Plot showing average plasma membrane ratios of detected annexin proteins upon digitonin treatment as compared to non-injured cells in MCF7-p95ErbB2 as measured by quantitative MS. Error bars represent SD for two independent

experiments. (**b**) Kinetics of ANXA7-RFP and Control-GFP translocation to damaged plasma membrane in response to focal laser injury in MCF7-p95ErbB2 cells. Error bars represent SD from six independent cells per condition. (**c**) Plasma membrane repair kinetics upon laser injury measured by membrane impermeable FM1-43 dye influx in MCF7-p95ErbB2 transfected with Ctrl, ANXA5, ANXA7 or ANXA5 and ANXA7 siRNAs for 72 h. Error bars represent SEM for at least 7 independent cells per condition. P-values based on Student's t-test: $*P \leq 0.05$, $**P \leq 0.01$

(**d**) Corresponding immunoblots showing protein levels of ANXA5, ANXA7 upon siRNA transfection (72 h) in MCF7-p95ErbB2 cells. HSP90 served as internal loading control (**e**) MCF7-p95ErbB2 cells transfected with indicated siRNAs for 72 h and treated with digitonin at the indicated concentrations for 1h at 37 °C. Plasma membrane integrity was measured by propidium iodide exclusion assay. Error bars indicate SD of three independent experiments. The asterisk represent P-values based on 2way Anova test: $**P \leq 0.01$, $***P \leq 0.001$, as indicated when comparing indicated siRNAs to control siRNA. (**f**) Complete blot for d.

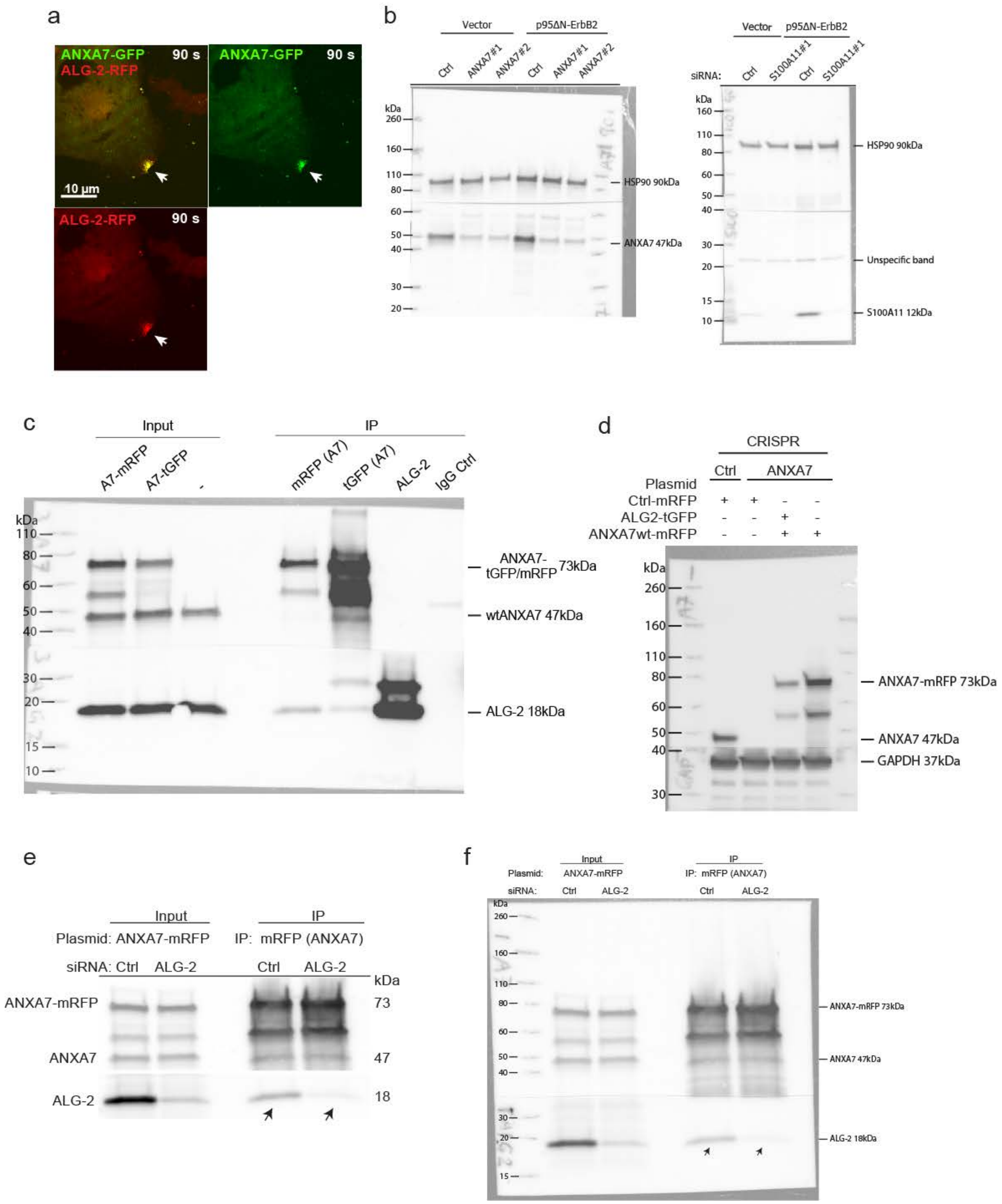

Supplementary Figure 2 (**a**) Extended confocal images (from Fig. 2a) of MCF7-p95ErbB2 cell expressing ALG-2-RFP and ANXA7-GFP captured after laser injury (90 s) shown in respective channels (white arrow indicates injury site) (**b**) Complete blot of Fig. 1f showing

protein levels of ANXA7 and S100A11 in MCF7 Vector Control and MCF7-p95ErbB2 cells. (**c**) Complete blot for Fig. 2c (**d**) Representative immunoblots showing protein levels in MCF7-p95ErbB2 Ctrl-CRISPR or A7-CRISPR cells 24h after transfection with ANXA7-RFP Ctrl-RFP or ANXA7-RFP and ALG-2-GFP. (**e**) Representative immunoprecipitation experiment using mRFP-antibody from lysates of MCF7-p95ErbB2 cells. Cells overexpressing ANXA7-mRFP were depleted for ALG-2 or not (Ctrl) by siRNA and exposed to scrape injury. Following IP, immunoblot analysis was carried out using ANXA7 and ALG-2 antibodies. (**f**) Complete blot of e.

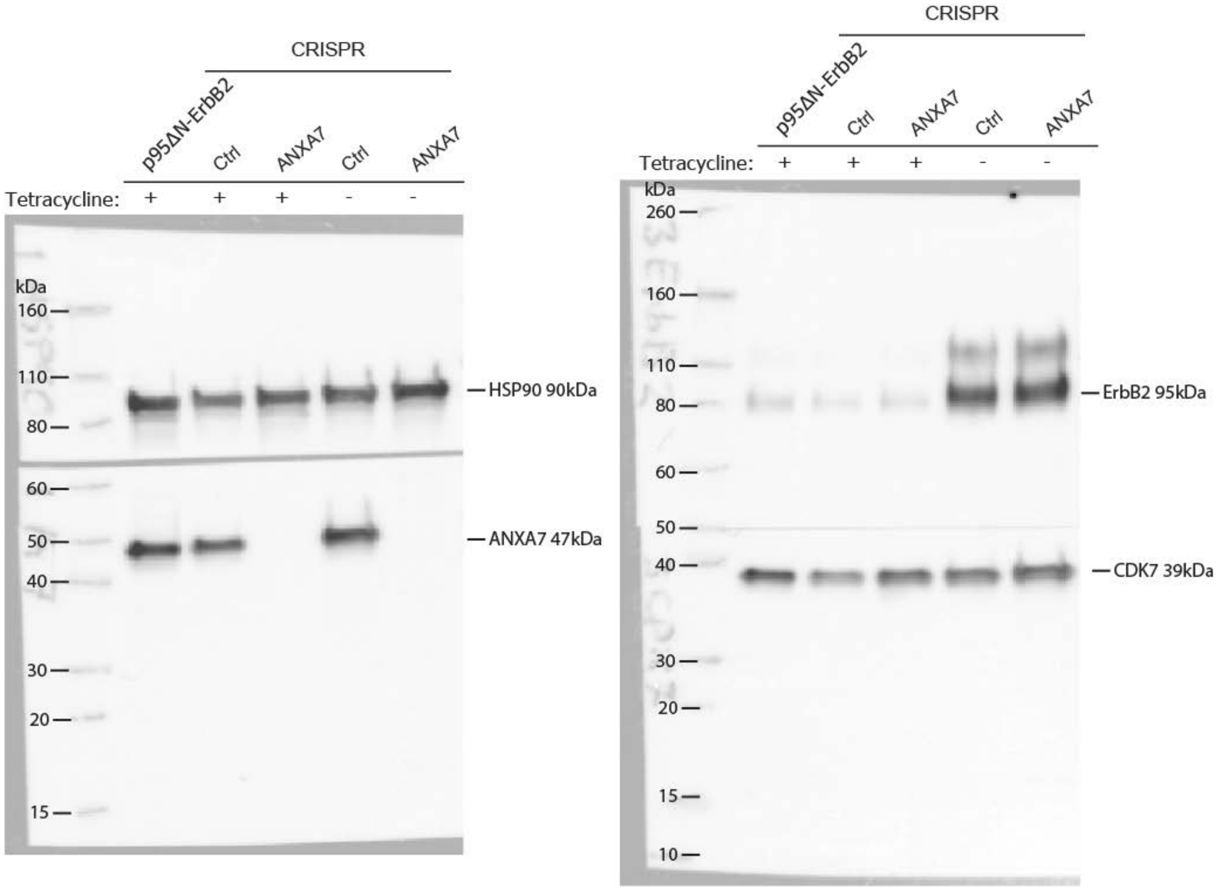

Supplementary Figure 3 Complete blot of Fig. 2f.

MOVIE LEGENDS

Supplementary Movie S1 ANXA7-RFP and ALG-2-GFP co-accumulate at the damaged membrane upon laser injury of MCF7-p95ErbB2 cell (Related to Fig. 2a). Scale bar, 10 µm.

Supplementary Movie S2 ANXA7 induces change in supported membrane conformation resulting in pearl-like structures in the patch periphery and contraction of membrane patch area (Related to Fig. 3b). Scale bar, 50 µm.

Supplementary Movie S3 ANXA7-RFP/ALG-2-GFP complex translocate to damaged membrane upon laser injury in MCF7-p95ErbB2 A7-CRISPR cell and trigger excision and shedding of membrane containing protrusions (Related to Fig. 4g). Scale bar, 3 µm.

Supplementary Movie S4 Representative Movie of ANXA7-RFP and ALG-2-GFP translocation and co-accumulation to damaged membrane upon laser injury in MCF7-p95ErbB2 A7-CRISPR cell and subsequent shedding of damaged membrane (Related to Fig. 4). Scale bar, 3 µm.

Supplementary Movie S5 Movie of ANXA7-RFP and ALG-2-GFP translocation and co-accumulation to damaged membrane after plasma membrane laser injury in MCF7-p95ErbB2 A7-CRISPR cell and subsequent shedding of damaged membrane (Related to Fig. 4a-c). Scale bar, 3 µm

Supplementary Table 1 Proteins associated with the plasma membrane in MCF7-p95ErbB2 cells exposed to digitonin as quantified by the SILAC method using MS.

Supplementary Table 1: Proteins associated with the plasma membrane in MCF7-p95ErbB2 cells as quantified by the SILAC method using MS.

No.	Protein ID	Protein name	Gene name	No. of proteins [a)]	Seq. Pep. [b)]	Seq. Cov. % [c)]	ErbB2-digi /ErbB2 [d)]	Var. % [e)]
1	Q9UKD2	mRNA turnover protein 4 homolog	MRTO4	1	3	15.5	4.368	128.32
2	Q99829	Copine-1	CPNE1	18	9	15.2	4.324	22.55
3	P46060	Ran GTPase-activating protein 1	RANGAP1	5	3	7.5	4.222	122.55
4	P08758	Annexin A5	ANXA5	5	23	69.7	3.864	51.81
5	O15091	Mitochondrial ribonuclease P protein 3	KIAA0391	4	3	6.9	3.696	121.84
6	P04083	Annexin A1	ANXA1	3	12	38.7	3.643	15.70
7	P20073	Annexin A7	ANXA7	3	15	43.2	3.346	36.24
8	P12429	Annexin A3	ANXA3	6	13	40.6	3.133	19.42
9	P09525	Annexin A4	ANXA4	3	16	48.2	2.696	19.74
10	O14929	Histone acetyltransferase type B catalytic subunit	HAT1	2	3	11.4	2.549	26.82
11	Q08209	Serine/threonine-protein phosphatase 2B catalytic subunit alpha isoform	PPP3CA	16	4	17.6	2.491	12.03
12	Q13616	Cullin-1	CUL1	1	2	2.4	2.371	96.90
13	O14979	Heterogeneous nuclear ribonucleoprotein D-like	HNRNPDL	3	3	13.9	2.355	148.50
14	P05455	Lupus La protein	SSB	4	13	31.4	2.340	12.15
15	P49748	Very long-chain specific acyl-CoA dehydrogenase, mitochondrial	ACADVL	11	16	31.4	2.301	23.62
16	Q9H1K4	Mitochondrial glutamate carrier 1	SLC25A22	12	3	12.2	2.279	5.70
17	P49321	Nuclear autoantigenic sperm protein	NASP	11	11	20.4	2.276	9.18
18	H7C466	Zinc finger CCCH domain-containing protein 15	ZC3H15	1	2	29.6	2.259	34.30
19	O60313	Dynamin-like 120 kDa protein, mitochondrial	OPA1	9	11	14.6	2.239	15.06
20	Q6NUK1	Calcium-binding mitochondrial carrier protein SCaMC-1	SLC25A24	3	15	36.0	2.214	27.39
21	Q9H3K2	Growth hormone-inducible transmembrane protein	GHITM	2	2	6.5	2.171	120.37
22	Q9NVI7	ATPase family AAA domain-containing protein 3A	ATAD3A	11	13	26.1	2.168	26.64
23	P57105	Synaptojanin-2-binding protein	SYNJ2BP	1	2	17.2	2.151	9.57
24	Q9Y6N5	Sulfide:quinone oxidoreductase, mitochondrial	SQRDL	6	10	24.9	2.150	27.76
25	P40939	Trifunctional enzyme subunit alpha, mitochondrial	HADHA	3	35	51.2	2.149	22.30
26	Q08J23	tRNA (cytosine(34)-C(5))-methyltransferase	NSUN2	3	19	27.2	2.149	29.50
27	Q96A26	Protein FAM162A	FAM162A	2	4	27.1	2.084	16.02
28	O95202	LETM1 and EF-hand domain-containing protein 1,	LETM1	3	16	27.2	2.081	41.58

Supplementary Table 1: Continued.

No.	Protein ID	Protein name	Gene name	No. of proteins a)	Seq. Pep. b)	Seq. Cov. % c)	ErbB2-digi /ErbB2 d)	Var. % e)
29	P27144	Adenylate kinase 4, mitochondrial	AK4	2	9	58.3	2.080	23.48
30	P43246	DNA mismatch repair protein Msh2	MSH2	4	6	8.3	2.069	44.36
31	P07384	Calpain-1 catalytic subunit	CAPN1	11	19	27.3	2.067	37.96
32	P24539	ATP synthase F(0) complex subunit B1, mitochondrial	ATP5F1	2	11	56.9	2.064	11.82
33	Q5RI15	Cytochrome c oxidase protein 20 homolog	COX20	2	2	21.2	2.054	45.31
34	Q9Y4W6	AFG3-like protein 2	AFG3L2	8	17	21.0	2.047	16.47
35	O15355	Protein phosphatase 1G	PPM1G	1	8	17.9	2.039	23.02
36	P54886	Delta-1-pyrroline-5-carboxylate synthase	ALDH18A1	2	24	35.4	2.014	22.54
37	Q16891	Mitochondrial inner membrane protein	IMMT	8	40	57.3	2.011	31.46
38	P22695	Cytochrome b-c1 complex subunit 2, mitochondrial	UQCRC2	6	13	37.7	2.008	31.95
39	O60701	UDP-glucose 6-dehydrogenase	UGDH	7	8	23.3	1.956	30.41
40	Q9NX63	Coiled-coil-helix-coiled-coil-helix domain-containing protein 3, mitochondrial	CHCHD3	5	8	28.6	1.955	13.13
41	Q9Y3D7	Mitochondrial import inner membrane translocase subunit TIM16	PAM16	5	2	27.2	1.952	15.14
42	P12235	ADP/ATP translocase 1	SLC25A4	2	11	30.2	1.910	1.56
43	O95299	NADH dehydrogenase [ubiquinone] 1 alpha subcomplex subunit 10, mitochondrial	NDUFA10	8	7	19.2	1.902	17.34
44	Q96QD9	UAP56-interacting factor	FYTTD1	7	2	8.2	1.901	105.38
45	O75131	Copine-3	CPNE3	20	15	26.6	1.870	33.65
46	P25705	ATP synthase subunit alpha, mitochondrial	ATP5A1	9	35	60.9	1.858	27.39
47	Q12931	Heat shock protein 75 kDa, mitochondrial	TRAP1	6	26	47.0	1.855	27.38
48	P42704	Leucine-rich PPR motif-containing protein, mitochondrial	LRPPRC	8	55	40.0	1.854	26.24
49	P20674	Cytochrome c oxidase subunit 5A, mitochondrial	COX5A	5	7	46.0	1.854	14.94
50	P31930	Cytochrome b-c1 complex subunit 1, mitochondrial	UQCRC1	1	10	26.9	1.843	26.54
51	Q00325	Phosphate carrier protein, mitochondrial	SLC25A3	6	9	19.1	1.839	16.92
52	P36542	ATP synthase subunit gamma, mitochondrial	ATP5C1	3	9	28.2	1.830	32.22
53	Q96BW9	Mitochondrial translocator assembly and maintenance protein 41 homolog	TAMM41	6	2	9.2	1.824	14.85
54	Q8IXI1	Mitochondrial Rho GTPase 2	RHOT2	16	4	8.4	1.814	13.46

Supplementary Table 1: Continued.

No.	Protein ID	Protein name	Gene name	No. of proteins a)	Seq. Pep. b)	Seq. Cov. % c)	ErbB2-digi /ErbB2 d)	Var. % e)
55	P35579	Myosin-9	MYH9	2	25	14.8	1.811	26.79
56	O95182	NADH dehydrogenase [ubiquinone] 1 alpha subcomplex subunit 7	NDUFA7	1	2	16.8	1.811	29.99
57	P55084	Trifunctional enzyme subunit beta, mitochondrial	HADHB	7	27	57.3	1.805	45.29
58	P07741	Adenine phosphoribosyltransferase	APRT	6	10	52.8	1.800	67.52
59	Q9UIJ7	GTP:AMP phosphotransferase AK3, mitochondrial	AK3	3	6	30.4	1.793	14.60
60	P47985	Cytochrome b-c1 complex subunit Rieske, mitochondrial	UQCRFS1	2	8	27.4	1.792	22.17
61	P43304	Glycerol-3-phosphate dehydrogenase, mitochondrial	GPD2	6	27	41.3	1.788	30.42
62	P56134	ATP synthase subunit f, mitochondrial	ATP5J2	8	2	49.0	1.774	13.34
63	O94925	Glutaminase kidney isoform, mitochondrial	GLS	4	6	14.7	1.749	35.90
64	P50995	Annexin A11	ANXA11	7	11	23.3	1.748	10.23
65	P49915	GMP synthase [glutamine-hydrolyzing]	GMPS	2	8	15.0	1.745	37.28
66	P12236	ADP/ATP translocase 3	SLC25A6	3	16	41.9	1.743	20.25
67	O00217	NADH dehydrogenase [ubiquinone] iron-sulfur protein 8, mitochondrial	NDUFS8	5	2	19.1	1.738	15.50
68	P48047	ATP synthase subunit O, mitochondrial	ATP5O	4	10	55.4	1.737	17.52
69	Q7L0Y3	Mitochondrial ribonuclease P protein 1	TRMT10C	2	3	9.7	1.728	8.84
70	P28331	NADH-ubiquinone oxidoreductase 75 kDa subunit, mitochondrial	NDUFS1	7	16	33.7	1.726	28.57
71	Q9HCC0	Methylcrotonoyl-CoA carboxylase beta chain, mitochondrial	MCCC2	5	17	36.1	1.725	19.44
72	Q9Y383	Putative RNA-binding protein Luc7-like 2	LUC7L2	4	2	6.9	1.724	15.79
73	Q96HS1	Serine/threonine-protein phosphatase PGAM5, mitochondrial	PGAM5	3	10	34.9	1.715	65.74
74	Q9UHQ9	NADH-cytochrome b5 reductase 1	CYB5R1	2	9	26.9	1.715	21.47
75	P10515	Dihydrolipoyllysine-residue acetyltransferase component of pyruvate dehydrogenase complex, mitochondrial	DLAT	4	7	15.9	1.712	19.76
76	P08574	Cytochrome c1, heme protein, mitochondrial	CYC1	1	8	31.1	1.711	36.70
77	Q9NQX4	Unconventional myosin-Vc	MYO5C	6	14	9.1	1.709	79.07

Supplementary Table 1: Continued.

No.	Protein ID	Protein name	Gene name	No. of proteins a)	Seq. Pep. b)	Seq. Cov. % c)	ErbB2-digi /ErbB2 d)	Var. % e)
78	Q9UJS0	Calcium-binding mitochondrial carrier protein Aralar2	SLC25A13	4	23	48.3	1.706	24.75
79	P06702	Protein S100-A9	S100A9	1	7	52.6	1.705	16.01
80	Q9NQG5	Regulation of nuclear pre-mRNA domain-containing protein 1B	RPRD1B	2	3	11.0	1.702	5.52
81	P30049	ATP synthase subunit delta, mitochondrial	ATP5D	1	3	17.3	1.701	6.92
82	P31040	Succinate dehydrogenase [ubiquinone] flavoprotein subunit, mitochondrial	SDHA	4	5	11.0	1.694	20.81
83	O75947	ATP synthase subunit d, mitochondrial	ATP5H	3	11	73.9	1.694	22.94
84	P05141	ADP/ATP translocase 2	SLC25A5	1	15	36.9	1.687	18.97
85	P13073	Cytochrome c oxidase subunit 4 isoform 1, mitochondrial	COX4I1	6	11	55.0	1.683	27.11
86	P51648	Fatty aldehyde dehydrogenase	ALDH3A2	7	4	13.7	1.682	146.17
87	O96000	NADH dehydrogenase [ubiquinone] 1 beta subcomplex subunit 10	NDUFB10	4	6	37.2	1.681	18.02
88	Q9BPW8	Protein NipSnap homolog 1	NIPSNAP1	4	4	21.8	1.681	25.31
89	P14927	Cytochrome b-c1 complex subunit 7	UQCRB	4	5	45.0	1.680	4.18
90	P52789	Hexokinase-2	HK2	5	16	19.0	1.668	11.67
91	P19367	Hexokinase-1	HK1	9	28	30.6	1.668	18.40
92	Q9Y3D9	28S ribosomal protein S23, mitochondrial	MRPS23	2	4	26.3	1.658	11.35
93	Q9Y2R9	28S ribosomal protein S7, mitochondrial	MRPS7	5	3	20.2	1.657	1.84
94	Q9Y2Q3	Glutathione S-transferase kappa 1	GSTK1	5	2	16.4	1.657	8.36
95	Q9P258	Protein RCC2	RCC2	1	7	13.4	1.654	14.62
96	P21796	Voltage-dependent anion-selective channel protein 1	VDAC1	2	17	69.6	1.654	25.17
97	Q9H845	Acyl-CoA dehydrogenase family member 9, mitochondrial	ACAD9	3	6	10.6	1.646	36.03
98	O94826	Mitochondrial import receptor subunit TOM70	TOMM70A	1	18	38.2	1.633	37.57
99	O43615	Mitochondrial import inner membrane translocase subunit TIM44	TIMM44	5	8	21.5	1.631	29.41
100	P31949	Protein S100-A11	S100A11	1	4	41.9	1.631	66.10
101	P06576	ATP synthase subunit beta, mitochondrial	ATP5B	6	28	75.4	1.625	23.59
102	P54819	Adenylate kinase 2, mitochondrial	AK2	11	11	65.9	1.616	25.57

Supplementary Table 1: Continued.

No.	Protein ID	Protein name	Gene name	No. of proteins [a)]	Seq. Pep. [b)]	Seq. Cov. % [c)]	ErbB2-digi /ErbB2 [d)]	Var. % [e)]
103	Q96RQ3	Methylcrotonoyl-CoA carboxylase subunit alpha, mitochondrial	MCCC1	6	18	31.0	1.612	23.54
104	P35232	Prohibitin	PHB	7	19	75.4	1.610	15.90
105	P62258	14-3-3 protein epsilon	YWHAE	7	15	54.9	1.610	102.08
106	O75431	Metaxin-2	MTX2	4	3	21.7	1.604	6.12
107	O95168	NADH dehydrogenase [ubiquinone] 1 beta subcomplex subunit 4	NDUFB4	4	5	48.1	1.597	98.93
108	P13010	X-ray repair cross-complementing protein 5	XRCC5	3	29	49.6	1.594	19.92
109	Q9NZT2	Opioid growth factor receptor	OGFR	2	3	8.1	1.593	26.16
110	P10606	Cytochrome c oxidase subunit 5B, mitochondrial	COX5B	1	5	35.7	1.590	13.63
111	P52701	DNA mismatch repair protein Msh6	MSH6	6	17	18.4	1.589	89.85
112	P17568	NADH dehydrogenase [ubiquinone] 1 beta subcomplex subunit 7	NDUFB7	1	3	19.7	1.589	22.16
113	P19404	NADH dehydrogenase [ubiquinone] flavoprotein 2, mitochondrial	NDUFV2	2	4	21.7	1.585	29.55
114	O43920	NADH dehydrogenase [ubiquinone] iron-sulfur protein 5	NDUFS5	1	2	17.9	1.583	0.52
115	P04632	Calpain small subunit 1	CAPNS1	10	8	55.6	1.575	34.94
116	P45880	Voltage-dependent anion-selective channel protein 2	VDAC2	7	9	36.4	1.568	10.73
117	Q6UB35	Monofunctional C1-tetrahydrofolate synthase, mitochondrial	MTHFD1L	10	7	8.4	1.563	17.95
118	P56556	NADH dehydrogenase [ubiquinone] 1 alpha subcomplex subunit 6	NDUFA6	2	3	22.7	1.556	9.24
119	Q9H9B4	Sideroflexin-1	SFXN1	5	12	44.1	1.553	33.65
120	O95831	Apoptosis-inducing factor 1, mitochondrial	AIFM1	7	19	37.9	1.550	31.46
121	Q9GZT3	SRA stem-loop-interacting RNA-binding protein, mitochondrial	SLIRP	7	4	46.7	1.547	23.62
122	O00267	Transcription elongation factor SPT5	SUPT5H	2	4	5.7	1.542	18.58
123	Q86VP6	Cullin-associated NEDD8-dissociated protein 1	CAND1	8	27	24.8	1.537	41.56
124	P53007	Tricarboxylate transport protein, mitochondrial	SLC25A1	2	7	23.2	1.530	24.83
125	P12956	X-ray repair cross-complementing protein 6	XRCC6	4	30	55.2	1.526	35.19

Supplementary Table 1: Continued.

No.	Protein ID	Protein name	Gene name	No. of proteins [a)]	Seq. Pep. [b)]	Seq. Cov. % [c)]	ErbB2-digi /ErbB2 [d)]	Var. % [e)]
126	P61313	60S ribosomal protein L15	RPL15	6	9	40.7	1.523	35.26
127	Q9Y2R0	Cytochrome c oxidase assembly factor 3 homolog, mitochondrial	COA3	2	5	47.2	1.522	22.81
128	Q96TA2	ATP-dependent zinc metalloprotease YME1L1	YME1L1	6	7	16.5	1.522	28.89
129	O14925	Mitochondrial import inner membrane translocase subunit Tim23	TIMM23	7	3	16.7	1.515	9.58
130	Q99623	Prohibitin-2	PHB2	9	18	63.9	1.511	16.55
131	Q9P2E9	Ribosome-binding protein 1	RRBP1	21	50	36.0	1.506	28.68
132	P00403	Cytochrome c oxidase subunit 2	MT-CO2	1	6	27.8	1.499	17.68
133	Q92621	Nuclear pore complex protein Nup205	NUP205	1	3	2.0	1.495	37.42
134	Q13268	Dehydrogenase/reductase SDR family member 2, mitochondrial	DHRS2	5	4	19.6	1.493	21.92
135	O76031	ATP-dependent Clp protease ATP-binding subunit clpX-like, mitochondrial	CLPX	1	9	17.5	1.493	19.31
136	P49411	Elongation factor Tu, mitochondrial	TUFM	2	19	49.8	1.490	22.58
137	O75489	NADH dehydrogenase [ubiquinone] iron-sulfur protein 3, mitochondrial	NDUFS3	5	7	35.6	1.487	8.29
138	Q5JRA6	Melanoma inhibitory activity protein 3	MIA3	5	17	14.2	1.484	74.09
139	P55210	Caspase-7	CASP7	2	2	6.3	1.483	36.45
140	Q05655	Protein kinase C delta type	PRKCD	5	8	11.1	1.476	15.31
141	P17655	Calpain-2 catalytic subunit	CAPN2	2	2	2.4	1.475	58.54
142	O00232	26S proteasome non-ATPase regulatory subunit 12	PSMD12	2	9	23.0	1.475	50.63
143	Q00059	Transcription factor A, mitochondrial	TFAM	3	8	31.7	1.475	24.12
144	Q6Y7W6	PERQ amino acid-rich with GYF domain-containing protein 2	GIGYF2	9	3	3.0	1.472	112.47
145	O15118	Niemann-Pick C1 protein	NPC1	3	2	1.9	1.472	15.84
146	P32322	Pyrroline-5-carboxylate reductase 1, mitochondrial	PYCR1	14	7	34.2	1.471	17.60
147	Q9UM54	Unconventional myosin-VI	MYO6	6	24	21.9	1.466	33.37
148	Q96C36	Pyrroline-5-carboxylate reductase 2	PYCR2	2	3	13.8	1.465	28.60
149	A0FGR8	Extended synaptotagmin-2	ESYT2	7	8	13.1	1.464	23.62
150	Q9BYN8	28S ribosomal protein S26, mitochondrial	MRPS26	1	3	18.0	1.462	9.10

Supplementary Table 1: Continued.

No.	Protein ID	Protein name	Gene name	No. of proteins a)	Seq. Pep. b)	Seq. Cov. % c)	ErbB2-digi /ErbB2 d)	Var. % e)
151	P11498	Pyruvate carboxylase, mitochondrial	PC	4	34	36.5	1.460	32.46
152	Q7L2E3	Putative ATP-dependent RNA helicase DHX30	DHX30	4	17	17.5	1.458	37.62
153	Q8TCJ2	Dolichyl-diphosphooligosaccharide--protein glycosyltransferase subunit STT3B	STT3B	1	3	4.4	1.447	16.84
154	Q14980	Nuclear mitotic apparatus protein 1	NUMA1	15	37	20.5	1.437	38.11
155	P22314	Ubiquitin-like modifier-activating enzyme 1	UBA1	7	29	32.5	1.433	38.10
156	P30825	High affinity cationic amino acid transporter 1	SLC7A1	2	2	3.8	1.433	34.35
157	P51151	Ras-related protein Rab-9A	RAB9A	2	2	12.4	1.428	36.16
158	Q14573	Inositol 1,4,5-trisphosphate receptor type 3	ITPR3	1	5	2.1	1.427	32.96
159	P33897	ATP-binding cassette sub-family D member 1	ABCD1	2	2	11.7	1.421	21.23
160	Q02338	D-beta-hydroxybutyrate dehydrogenase, mitochondrial	BDH1	4	2	14.1	1.419	42.89
161	P40429	60S ribosomal protein L13a	RPL13A	4	7	29.6	1.413	29.92
162	Q12789	General transcription factor 3C polypeptide 1	GTF3C1	4	5	3.0	1.406	49.33
163	P27695	DNA-(apurinic or apyrimidinic site) lyase	APEX1	9	9	43.8	1.404	15.57
164	P45974	Ubiquitin carboxyl-terminal hydrolase 5	USP5	6	13	23.6	1.404	39.11
165	Q9Y2Q9	28S ribosomal protein S28, mitochondrial	MRPS28	3	2	12.3	1.403	4.73
166	Q9Y6C9	Mitochondrial carrier homolog 2	MTCH2	2	4	17.5	1.402	19.50
167	P82650	28S ribosomal protein S22, mitochondrial	MRPS22	7	3	14.1	1.400	43.63
168	Q9NX40	OCIA domain-containing protein 1	OCIAD1	3	2	10.1	1.400	13.77
169	Q13423	NAD(P) transhydrogenase, mitochondrial	NNT	6	19	22.7	1.398	35.88
170	Q9H7Z7	Prostaglandin E synthase 2	PTGES2	3	9	34.5	1.396	25.91
171	O60664	Perilipin-3	PLIN3	7	9	30.6	1.385	18.59
172	Q92552	28S ribosomal protein S27, mitochondrial	MRPS27	4	3	14.4	1.378	12.87
173	Q9BUF5	Tubulin beta-6 chain	TUBB6	10	13	33.9	1.371	37.02
174	Q99714	3-hydroxyacyl-CoA dehydrogenase type-2	HSD17B10	3	7	44.8	1.366	14.95
175	P61086	Ubiquitin-conjugating enzyme E2 K	UBE2K	6	4	28.1	1.361	59.38
176	O96008	Mitochondrial import receptor subunit TOM40 homolog	TOMM40	4	7	32.4	1.359	14.23
177	Q07020	60S ribosomal protein L18	RPL18	8	8	37.2	1.359	15.38
178	P36776	Lon protease homolog, mitochondrial	LONP1	9	15	22.6	1.357	30.02

Supplementary Table 1: Continued.

No.	Protein ID	Protein name	Gene name	No. of proteins a)	Seq. Pep. b)	Seq. Cov. % c)	ErbB2-digi /ErbB2 d)	Var. % e)
179	Q70UQ0	Inhibitor of nuclear factor kappa-B kinase-interacting protein	IKBIP	1	4	11.7	1.349	1.52
180	Q6NUQ4	Transmembrane protein 214	TMEM214	6	5	8.2	1.349	14.48
181	Q9Y277	Voltage-dependent anion-selective channel protein 3	VDAC3	7	5	24.0	1.347	38.92
182	Q9BZZ5	Apoptosis inhibitor 5	API5	9	9	24.7	1.346	11.84
183	P62906	60S ribosomal protein L10a	RPL10A	1	8	27.6	1.342	13.71
184	O75165	DnaJ homolog subfamily C member 13	DNAJC13	2	8	4.5	1.342	13.71
185	Q02978	Mitochondrial 2-oxoglutarate/malate carrier protein	SLC25A11	3	7	33.8	1.340	30.65
186	Q969V3	Nicalin	NCLN	6	6	12.8	1.339	20.92
187	O15269	Serine palmitoyltransferase 1	SPTLC1	2	9	25.6	1.339	38.94
188	Q86U42	Polyadenylate-binding protein 2	PABPN1	6	5	31.1	1.338	27.83
189	P13674	Prolyl 4-hydroxylase subunit alpha-1	P4HA1	3	7	15.7	1.336	28.82
190	P63244	Guanine nucleotide-binding protein subunit beta-2-like 1	GNB2L1	22	11	30.3	1.334	15.45
191	P19338	Nucleolin	NCL	6	31	35.9	1.333	37.75
192	Q12965	Unconventional myosin-Ie	MYO1E	2	6	6.2	1.332	57.34
193	Q9H6F5	Coiled-coil domain-containing protein 86	CCDC86	2	3	6.7	1.330	52.38
194	Q8N183	Mimitin, mitochondrial	NDUFAF2	1	3	28.4	1.330	5.40
195	P62987	Ubiquitin	UBB	26	5	50.5	1.330	26.53
196	Q16718	NADH dehydrogenase [ubiquinone] 1 alpha subcomplex subunit 5	NDUFA5	5	3	40.2	1.329	10.81
197	Q9Y3E5	Peptidyl-tRNA hydrolase 2, mitochondrial	PTRH2	2	5	43.0	1.329	23.15
198	Q6I9Y2	THO complex subunit 7 homolog	THOC7	1	3	14.7	1.328	4.96
199	P25398	40S ribosomal protein S12	RPS12	1	3	30.3	1.328	7.33
200	P18077	60S ribosomal protein L35a	RPL35A	4	8	40.9	1.327	6.34
201	P62269	40S ribosomal protein S18	RPS18	2	15	63.2	1.326	16.74
202	O43143	Putative pre-mRNA-splicing factor ATP-dependent RNA helicase DHX15	DHX15	1	14	19.2	1.316	29.07
203	Q9NR28	Diablo homolog, mitochondrial	DIABLO	5	2	15.8	1.308	45.09
204	Q9UL46	Proteasome activator complex subunit 2	PSME2	3	9	41.4	1.308	9.44
205	P37268	Squalene synthase	FDFT1	7	4	13.1	1.308	5.85

Supplementary Table 1: Continued.

No.	Protein ID	Protein name	Gene name	No. of proteins a)	Seq. Pep. b)	Seq. Cov. % c)	ErbB2-digi /ErbB2 d)	Var. % e)
206	P40763	Signal transducer and activator of transcription 3	STAT3	4	5	10.4	1.301	9.45
207	Q9BW60	Elongation of very long chain fatty acids protein 1	ELOVL1	2	2	9.9	1.300	17.37
208	Q9Y6M9	NADH dehydrogenase [ubiquinone] 1 beta subcomplex subunit 9	NDUFB9	4	3	25.0	1.297	35.92
209	Q9BSJ8	Extended synaptotagmin-1	ESYT1	3	21	25.5	1.296	34.07
210	P30837	Aldehyde dehydrogenase X, mitochondrial	ALDH1B1	1	2	5.4	1.293	8.45
211	P02786	Transferrin receptor protein 1	TFRC	8	26	39.6	1.293	28.10
212	P26373	60S ribosomal protein L13	RPL13	5	10	45.5	1.292	27.18
213	Q8TCS8	Polyribonucleotide nucleotidyltransferase 1, mitochondrial	PNPT1	2	7	11.5	1.292	15.16
214	P46778	60S ribosomal protein L21	RPL21	3	9	42.5	1.290	29.05
215	Q9UBV2	Protein sel-1 homolog 1	SEL1L	3	3	5.8	1.287	15.56
216	O94905	Erlin-2	ERLIN2	6	8	26.3	1.284	13.76
217	Q9BUN8	Derlin-1	DERL1	4	3	17.9	1.284	8.98
218	P17252	Protein kinase C alpha type	PRKCA	4	2	5.7	1.279	53.17
219	P32969	60S ribosomal protein L9	RPL9	5	7	55.2	1.275	57.20
220	P49588	Alanine--tRNA ligase, cytoplasmic	AARS	2	29	38.6	1.272	25.75
221	Q14997	Proteasome activator complex subunit 4	PSME4	5	18	12.0	1.268	53.74
222	Q12849	G-rich sequence factor 1	GRSF1	6	2	10.4	1.263	14.20
223	Q7Z2K6	Endoplasmic reticulum metallopeptidase 1	ERMP1	3	5	5.9	1.262	15.76
224	P43307	Translocon-associated protein subunit alpha	SSR1	7	3	12.8	1.259	5.66
225	P67812	Signal peptidase complex catalytic subunit SEC11A	SEC11A	9	5	30.1	1.257	42.12
226	Q7Z6Z7	E3 ubiquitin-protein ligase HUWE1	HUWE1	6	10	3.4	1.255	19.60
227	Q13085	Acetyl-CoA carboxylase 1	ACACA	19	33	16.0	1.255	61.44
228	P10809	60 kDa heat shock protein, mitochondrial	HSPD1	8	45	72.1	1.252	39.67
229	P51659	Peroxisomal multifunctional enzyme type 2	HSD17B4	8	14	25.1	1.251	49.79
230	P00367	Glutamate dehydrogenase 1, mitochondrial	GLUD1	4	5	13.9	1.247	10.97
231	O75340	Programmed cell death protein 6	PDCD6	5	7	49.2	1.246	13.79
232	Q14566	DNA replication licensing factor MCM6	MCM6	1	22	29.5	1.246	36.76
233	P27635	60S ribosomal protein L10	RPL10	9	7	33.1	1.244	19.34
234	P23396	40S ribosomal protein S3	RPS3	14	21	72.0	1.242	65.98

Supplementary Table 1: Continued.

No.	Protein ID	Protein name	Gene name	No. of proteins a)	Seq. Pep. b)	Seq. Cov. % c)	ErbB2-digi /ErbB2 d)	Var. % e)
235	Q9BYD6	39S ribosomal protein L1, mitochondrial	MRPL1	3	3	13.2	1.241	38.71
236	P39019	40S ribosomal protein S19	RPS19	5	15	66.2	1.241	28.10
237	P35270	Sepiapterin reductase	SPR	1	5	29.5	1.241	7.21
238	O15260	Surfeit locus protein 4	SURF4	4	2	17.6	1.239	31.24
239	O95573	Long-chain-fatty-acid--CoA ligase 3	ACSL3	8	20	33.5	1.238	32.32
240	P11387	DNA topoisomerase 1	TOP1	7	23	29.2	1.238	34.67
241	Q9HAV7	GrpE protein homolog 1, mitochondrial	GRPEL1	1	4	21.7	1.236	11.80
242	Q16630	Cleavage and polyadenylation specificity factor subunit 6	CPSF6	5	6	18.0	1.236	39.70
243	Q9Y3A5	Ribosome maturation protein SBDS	SBDS	2	6	25.2	1.235	28.51
244	O14646	Chromodomain-helicase-DNA-binding protein 1	CHD1	11	3	2.8	1.235	51.06
245	Q6PIU2	Neutral cholesterol ester hydrolase 1	NCEH1	5	5	20.3	1.235	12.94
246	Q9HCU4	Cadherin EGF LAG seven-pass G-type receptor 2	CELSR2	1	3	1.3	1.234	0.98
247	P62913	60S ribosomal protein L11	RPL11	3	9	38.2	1.233	16.36
248	P29401	Transketolase	TKT	6	29	49.4	1.233	16.61
249	O75643	U5 small nuclear ribonucleoprotein 200 kDa helicase	SNRNP200	4	48	25.9	1.233	38.34
250	P62917	60S ribosomal protein L8	RPL8	5	8	25.7	1.231	19.66
251	P39023	60S ribosomal protein L3	RPL3	7	9	26.6	1.230	9.85
252	Q9Y4I1	Unconventional myosin-Va	MYO5A	10	14	9.0	1.226	24.06
253	P08865	40S ribosomal protein SA	RPSA	4	12	56.7	1.224	17.40
254	Q93050	V-type proton ATPase 116 kDa subunit a isoform 1	ATP6V0A1	12	3	3.9	1.224	28.29
255	Q15070	Mitochondrial inner membrane protein OXA1L	OXA1L	5	2	8.4	1.222	50.13
256	P49207	60S ribosomal protein L34	RPL34	1	5	22.2	1.220	3.62
257	Q96QK1	Vacuolar protein sorting-associated protein 35	VPS35	3	11	18.8	1.219	55.13
258	Q3ZCQ8	Mitochondrial import inner membrane translocase subunit TIM50	TIMM50	12	8	28.9	1.218	22.85
259	Q5JTV8	Torsin-1A-interacting protein 1	TOR1AIP1	6	4	8.9	1.214	73.46
260	P57088	Transmembrane protein 33	TMEM33	3	3	14.0	1.213	4.30
261	P31689	DnaJ homolog subfamily A member 1	DNAJA1	3	7	31.0	1.213	30.85
262	P50914	60S ribosomal protein L14	RPL14	2	4	17.7	1.213	17.50
263	P43897	Elongation factor Ts, mitochondrial	TSFM	8	4	28.8	1.212	30.62

Supplementary Table 1: Continued.

No.	Protein ID	Protein name	Gene name	No. of proteins a)	Seq. Pep. b)	Seq. Cov. % c)	ErbB2-digi /ErbB2 d)	Var. % e)
264	P38646	Stress-70 protein, mitochondrial	HSPA9	5	29	47.7	1.212	25.46
265	Q13724	Mannosyl-oligosaccharide glucosidase	MOGS	5	20	33.9	1.210	31.15
266	P62888	60S ribosomal protein L30	RPL30	3	4	18.4	1.210	4.82
267	Q7Z4W1	L-xylulose reductase	DCXR	7	2	10.3	1.206	11.24
268	P18124	60S ribosomal protein L7	RPL7	4	15	45.2	1.201	26.05
269	P36957	Dihydrolipoyllysine-residue succinyltransferase component of 2-oxoglutarate dehydrogenase complex, mitochondrial	DLST	6	6	14.6	1.201	5.87
270	O00410	Importin-5	IPO5	19	8	9.6	1.198	35.96
271	Q96N66	Lysophospholipid acyltransferase 7	MBOAT7	3	2	6.0	1.198	20.83
272	P35249	Replication factor C subunit 4	RFC4	8	6	20.1	1.198	28.26
273	Q00839	Heterogeneous nuclear ribonucleoprotein U	HNRNPU	3	33	32.4	1.196	55.40
274	Q9UJZ1	Stomatin-like protein 2, mitochondrial	STOML2	3	10	41.6	1.195	21.67
275	Q9NYU2	UDP-glucose:glycoprotein glucosyltransferase 1	UGGT1	5	21	16.3	1.195	61.23
276	O60216	Double-strand-break repair protein rad21 homolog	RAD21	1	2	3.6	1.192	65.49
277	Q8NI27	THO complex subunit 2	THOC2	4	9	6.8	1.192	69.42
278	Q9Y282	Endoplasmic reticulum-Golgi intermediate compartment protein 3	ERGIC3	8	3	32.3	1.190	40.78
279	Q7L014	Probable ATP-dependent RNA helicase DDX46	DDX46	2	6	6.2	1.190	6.26
280	Q92620	Pre-mRNA-splicing factor ATP-dependent RNA helicase PRP16	DHX38	6	6	6.1	1.189	39.53
281	Q5JPE7	Nodal modulator 2	NOMO2	6	17	18.1	1.188	24.54
282	Q00341	Vigilin	HDLBP	22	36	32.3	1.187	39.93
283	Q14435	Polypeptide N-acetylgalactosaminyltransferase 3	GALNT3	5	5	6.8	1.186	5.92
284	P46779	60S ribosomal protein L28	RPL28	8	9	46.7	1.184	22.86
285	Q6P2Q9	Pre-mRNA-processing-splicing factor 8	PRPF8	4	53	25.5	1.183	34.20
286	P42224	Signal transducer and activator of transcription 1-alpha/beta	STAT1	6	8	14.5	1.183	7.62
287	Q96A33	Coiled-coil domain-containing protein 47	CCDC47	3	20	42.0	1.181	21.51
288	Q9P035	Very-long-chain (3R)-3-hydroxyacyl-[acyl-carrier protein] dehydratase 3	PTPLAD1	6	3	7.1	1.180	20.90

Supplementary Table 1: Continued.

No.	Protein ID	Protein name	Gene name	No. of proteins a)	Seq. Pep. b)	Seq. Cov. % c)	ErbB2-digi /ErbB2 d)	Var. % e)
289	P61081	NEDD8-conjugating enzyme Ubc12	UBE2M	2	4	20.8	1.177	5.59
290	Q9UGP8	Translocation protein SEC63 homolog	SEC63	2	10	18.4	1.177	23.57
291	Q9NQC3	Reticulon-4	RTN4	9	7	38.1	1.173	88.40
292	Q8NFQ8	Torsin-1A-interacting protein 2	TOR1AIP2	1	5	14.7	1.172	16.61
293	P35250	Replication factor C subunit 2	RFC2	9	3	12.2	1.170	4.37
294	P16615	Sarcoplasmic/endoplasmic reticulum calcium ATPase 2	ATP2A2	10	18	23.6	1.167	28.00
295	Q13263	Transcription intermediary factor 1-beta	TRIM28	5	26	43.7	1.166	43.54
296	P62241	40S ribosomal protein S8	RPS8	2	9	43.6	1.161	12.99
297	P62249	40S ribosomal protein S16	RPS16	6	11	57.5	1.160	30.85
298	P04843	Dolichyl-diphosphooligosaccharide--protein glycosyltransferase subunit 1	RPN1	3	27	49.3	1.160	21.06
299	Q9NTZ6	RNA-binding protein 12	RBM12	4	4	5.5	1.158	0.43
300	Q92667	A-kinase anchor protein 1, mitochondrial	AKAP1	4	4	30.9	1.156	19.54
301	P82663	28S ribosomal protein S25, mitochondrial	MRPS25	4	2	12.7	1.155	19.73
302	P30519	Heme oxygenase 2	HMOX2	9	13	53.8	1.155	14.23
303	Q8IXB1	DnaJ homolog subfamily C member 10	DNAJC10	2	3	4.0	1.155	2.33
304	P51571	Translocon-associated protein subunit delta	SSR4	2	5	31.2	1.154	16.84
305	P50416	Carnitine O-palmitoyltransferase 1, liver isoform	CPT1A	6	16	24.3	1.153	37.08
306	P07355	Annexin A2	ANXA2	25	33	64.6	1.152	34.26
307	P06493	Cyclin-dependent kinase 1	CDK1	37	7	24.6	1.151	93.14
308	P61353	60S ribosomal protein L27	RPL27	4	10	54.4	1.151	36.98
309	O43809	Cleavage and polyadenylation specificity factor subunit 5	NUDT21	3	9	41.9	1.151	31.55
310	P38919	Eukaryotic initiation factor 4A-III	EIF4A3	2	21	46.5	1.150	27.59
311	P33991	DNA replication licensing factor MCM4	MCM4	7	19	29.1	1.148	13.55
312	P30050	60S ribosomal protein L12	RPL12	2	5	40.6	1.148	35.61
313	P36578	60S ribosomal protein L4	RPL4	4	17	36.1	1.145	32.01
314	P51572	B-cell receptor-associated protein 31	BCAP31	7	20	52.8	1.144	28.43
315	P05387	60S acidic ribosomal protein P2	RPLP2	3	11	97.4	1.144	29.63
316	P00533	Epidermal growth factor receptor	EGFR	7	7	8.2	1.143	29.31
317	Q9NZM1	Myoferlin	MYOF	28	63	34.0	1.140	49.75

Supplementary Table 1: Continued.

No.	Protein ID	Protein name	Gene name	No. of proteins a)	Seq. Pep. b)	Seq. Cov. % c)	ErbB2-digi /ErbB2 d)	Var. % e)
318	Q00610	Clathrin heavy chain 1	CLTC	11	61	42.1	1.139	36.73
319	Q9H307	Pinin	PNN	2	7	11.9	1.139	24.84
320	P60468	Protein transport protein Sec61 subunit beta	SEC61B	2	3	37.5	1.137	9.85
321	Q9BVK6	Transmembrane emp24 domain-containing protein 9	TMED9	1	9	27.7	1.136	32.01
322	P06733	Alpha-enolase	ENO1	15	25	53.7	1.134	60.52
323	P52815	Mitochondrial dicarboxylate carrier	SLC25A10	4	7	24.4	1.133	21.69
324	Q13247	Serine/arginine-rich splicing factor 6	SRSF6	4	9	23.3	1.133	35.98
325	P08648	Integrin alpha-5	ITGA5	2	6	7.9	1.133	25.42
326	Q02878	60S ribosomal protein L6	RPL6	7	16	45.5	1.132	19.73
327	Q14554	Protein disulfide-isomerase A5	PDIA5	2	3	8.3	1.131	21.25
328	O60341	Lysine-specific histone demethylase 1A	KDM1A	4	4	6.1	1.131	25.20
329	Q8NF37	Lysophosphatidylcholine acyltransferase 1	LPCAT1	1	6	12.4	1.130	24.39
330	O95861	3(2),5-bisphosphate nucleotidase 1	BPNT1	9	4	18.8	1.129	15.98
331	Q9UMS4	Pre-mRNA-processing factor 19	PRPF19	5	8	20.0	1.129	47.16
332	Q9UHG3	Prenylcysteine oxidase 1	PCYOX1	7	7	17.4	1.128	23.34
333	P62851	40S ribosomal protein S25	RPS25	1	6	31.2	1.127	12.03
334	Q9Y3D6	Mitochondrial fission 1 protein	FIS1	1	3	26.3	1.127	0.18
335	Q15029	116 kDa U5 small nuclear ribonucleoprotein component	EFTUD2	5	23	30.6	1.127	37.28
336	Q13510	Acid ceramidase	ASAH1	5	2	7.5	1.122	15.69
337	P11717	Cation-independent mannose-6-phosphate receptor	IGF2R	2	7	2.9	1.121	10.18
338	P51532	Transcription activator BRG1	SMARCA4	16	20	16.1	1.120	28.30
339	Q13618	Cullin-3	CUL3	5	3	5.4	1.120	34.73
340	P39656	Dolichyl-diphosphooligosaccharide--protein glycosyltransferase 48 kDa subunit	DDOST	3	12	42.1	1.119	31.29
341	Q9BTZ2	Dehydrogenase/reductase SDR family member 4	DHRS4	12	4	13.7	1.119	11.17
342	Q9NX14	NADH dehydrogenase [ubiquinone] 1 beta subcomplex subunit 11, mitochondrial	NDUFB11	2	2	22.2	1.119	41.54
343	P56545	C-terminal-binding protein 2	CTBP2	10	5	12.8	1.116	74.58
344	P20645	Cation-dependent mannose-6-phosphate receptor	M6PR	3	2	12.6	1.115	19.87
345	Q9HDC9	Adipocyte plasma membrane-associated protein	APMAP	3	17	46.4	1.115	49.89

Supplementary Table 1: Continued.

No.	Protein ID	Protein name	Gene name	No. of proteins [a)]	Seq. Pep. [b)]	Seq. Cov. % [c)]	ErbB2-digi /ErbB2 [d)]	Var. % [e)]
346	Q02543	60S ribosomal protein L18a	RPL18A	5	7	31.9	1.115	39.57
347	P09497	Clathrin light chain B	CLTB	3	4	16.6	1.114	5.78
348	Q9BTV4	Transmembrane protein 43	TMEM43	2	11	36.0	1.113	10.57
349	Q96CW1	AP-2 complex subunit mu	AP2M1	8	5	13.2	1.113	43.41
350	O96019	Actin-like protein 6A	ACTL6A	4	4	16.5	1.112	17.55
351	O95602	DNA-directed RNA polymerase I subunit RPA1	POLR1A	2	3	2.2	1.111	16.26
352	P30876	DNA-directed RNA polymerase II subunit RPB2	POLR2B	3	3	4.1	1.111	27.36
353	O14980	Exportin-1	XPO1	10	13	14.0	1.110	33.83
354	Q9Y3A6	Transmembrane emp24 domain-containing protein 5	TMED5	4	2	8.3	1.108	5.57
355	Q9BWH2	FUN14 domain-containing protein 2	FUNDC2	1	2	5.3	1.107	10.68
356	O43760	Synaptogyrin-2	SYNGR2	6	4	17.0	1.107	46.48
357	O43172	U4/U6 small nuclear ribonucleoprotein Prp4	PRPF4	2	6	16.1	1.106	15.57
358	Q8N201	Integrator complex subunit 1	INTS1	2	2	1.4	1.106	5.93
359	Q15428	Splicing factor 3A subunit 2	SF3A2	2	5	13.6	1.104	31.68
360	O95232	Luc7-like protein 3	LUC7L3	9	3	23.4	1.104	2.23
361	Q9Y230	RuvB-like 2	RUVBL2	6	15	34.8	1.103	40.06
362	P18206	Vinculin	VCL	5	24	29.6	1.101	74.72
363	Q8N138	ORM1-like protein 3	ORMDL3	4	3	24.1	1.100	23.39
364	P37108	Signal recognition particle 14 kDa protein	SRP14	2	9	44.9	1.099	27.26
365	O14735	CDP-diacylglycerol--inositol 3-phosphatidyltransferase	CDIPT	4	2	12.5	1.099	12.65
366	Q8NC51	Plasminogen activator inhibitor 1 RNA-binding protein	SERBP1	4	12	35.9	1.098	32.70
367	Q8IWX8	Calcium homeostasis endoplasmic reticulum protein	CHERP	2	5	7.8	1.098	17.28
368	Q9Y394	Dehydrogenase/reductase SDR family member 7	DHRS7	4	4	18.0	1.098	18.58
369	Q86Y56	HEAT repeat-containing protein 2	HEATR2	5	3	5.9	1.098	20.40
370	P46977	Dolichyl-diphosphooligosaccharide--protein glycosyltransferase subunit STT3A	STT3A	5	8	10.8	1.097	16.97
371	Q09028	Histone-binding protein RBBP4	RBBP4	12	8	20.7	1.097	11.87
372	P33993	DNA replication licensing factor MCM7	MCM7	4	22	37.1	1.097	19.81
373	Q9NR30	Nucleolar RNA helicase 2	DDX21	3	13	21.7	1.096	29.39
374	P22392	Nucleoside diphosphate kinase B	NME2	9	17	76.8	1.093	148.67
375	P60866	40S ribosomal protein S20	RPS20	5	5	26.1	1.093	12.14

Supplementary Table 1: Continued.

No.	Protein ID	Protein name	Gene name	No. of proteins a)	Seq. Pep. b)	Seq. Cov. % c)	ErbB2-digi /ErbB2 d)	Var. % e)
376	P25685	DnaJ homolog subfamily B member 1	DNAJB1	9	8	33.5	1.090	17.49
377	P11279	Lysosome-associated membrane glycoprotein 1	LAMP1	2	4	10.6	1.090	10.06
378	Q8TEM1	Nuclear pore membrane glycoprotein 210	NUP210	2	16	10.7	1.088	28.21
379	P00387	NADH-cytochrome b5 reductase 3	CYB5R3	4	6	32.6	1.088	26.54
380	Q09666	Neuroblast differentiation-associated protein AHNAK	AHNAK	7	163	46.0	1.088	36.15
381	Q13769	THO complex subunit 5 homolog	THOC5	5	4	22.5	1.088	6.22
382	Q8N684	Cleavage and polyadenylation specificity factor subunit 7	CPSF7	7	4	16.3	1.088	4.22
383	P08579	U2 small nuclear ribonucleoprotein B	SNRPB2	1	7	35.6	1.087	8.25
384	P16435	NADPH--cytochrome P450 reductase	POR	8	26	43.4	1.086	35.53
385	Q14165	Malectin	MLEC	4	6	29.5	1.085	11.16
386	Q8IWS0	PHD finger protein 6	PHF6	6	3	13.0	1.084	15.70
387	P48449	Lanosterol synthase	LSS	4	7	13.0	1.084	39.40
388	Q9P0I2	ER membrane protein complex subunit 3	EMC3	3	4	29.2	1.083	8.87
389	Q9UM00	Transmembrane and coiled-coil domain-containing protein 1	TMCO1	5	3	35.6	1.083	10.57
390	O60264	SWI/SNF-related matrix-associated actin-dependent regulator of chromatin subfamily A member 5	SMARCA5	3	10	9.5	1.082	50.16
391	P08621	U1 small nuclear ribonucleoprotein 70 kDa	SNRNP70	6	6	15.8	1.081	119.42
392	Q15233	Non-POU domain-containing octamer-binding protein	NONO	6	21	45.9	1.081	27.97
393	Q15155	Nodal modulator 1	NOMO1	3	17	17.7	1.080	31.97
394	P22102	Trifunctional purine biosynthetic protein adenosine-3	GART	9	12	13.7	1.078	14.49
395	O75955	Flotillin-1	FLOT1	8	9	28.2	1.077	31.43
396	Q15904	V-type proton ATPase subunit S1	ATP6AP1	6	4	10.9	1.076	3.97
397	Q92896	Golgi apparatus protein 1	GLG1	6	7	6.5	1.074	21.35
398	P24928	DNA-directed RNA polymerase II subunit RPB1	POLR2A	2	8	7.0	1.074	29.76
399	P60903	Protein S100-A10	S100A10	1	2	27.8	1.074	14.90
400	Q13509	Tubulin beta-3 chain	TUBB3	9	18	49.3	1.073	43.45
401	Q9NTJ5	Phosphatidylinositide phosphatase SAC1	SACM1L	6	4	6.1	1.070	38.62
402	O00391	Sulfhydryl oxidase 1	QSOX1	3	2	4.8	1.069	4.03
403	Q9Y679	Ancient ubiquitous protein 1	AUP1	3	3	11.3	1.069	20.07

Supplementary Table 1: Continued.

No.	Protein ID	Protein name	Gene name	No. of proteins a)	Seq. Pep. b)	Seq. Cov. % c)	ErbB2-digi /ErbB2 d)	Var. % e)
404	P13639	Elongation factor 2	EEF2	1	40	46.4	1.069	29.18
405	Q96T76	MMS19 nucleotide excision repair protein homolog	MMS19	7	2	2.3	1.069	52.35
406	P46777	60S ribosomal protein L5	RPL5	3	10	28.6	1.069	17.83
407	Q8TC12	Retinol dehydrogenase 11	RDH11	8	5	21.7	1.068	20.33
408	Q8WWY3	U4/U6 small nuclear ribonucleoprotein Prp31	PRPF31	8	5	14.6	1.067	23.88
409	Q9BUQ8	Probable ATP-dependent RNA helicase DDX23	DDX23	3	10	12.8	1.067	25.56
410	P62424	60S ribosomal protein L7a	RPL7A	3	13	33.8	1.067	36.60
411	Q07065	Cytoskeleton-associated protein 4	CKAP4	1	24	48.8	1.066	25.65
412	P25205	DNA replication licensing factor MCM3	MCM3	4	22	36.8	1.066	25.78
413	P05386	60S acidic ribosomal protein P1	RPLP1	1	2	28.9	1.066	5.97
414	O75934	Pre-mRNA-splicing factor SPF27	BCAS2	1	4	32.9	1.063	18.92
415	O95373	Importin-7	IPO7	2	9	10.1	1.063	25.30
416	P04844	Dolichyl-diphosphooligosaccharide--protein glycosyltransferase subunit 2	RPN2	7	14	31.7	1.062	22.42
417	Q86UE4	Protein LYRIC	MTDH	5	13	31.1	1.060	61.05
418	P52209	6-phosphogluconate dehydrogenase, decarboxylating	PGD	7	14	34.5	1.060	9.62
419	Q16698	2,4-dienoyl-CoA reductase, mitochondrial	DECR1	7	6	22.7	1.060	16.51
420	O60832	H/ACA ribonucleoprotein complex subunit 4	DKC1	5	3	7.8	1.058	70.50
421	Q04837	Single-stranded DNA-binding protein, mitochondrial	SSBP1	3	4	33.1	1.056	11.04
422	P17844	Probable ATP-dependent RNA helicase DDX5	DDX5	14	36	47.9	1.056	29.41
423	Q9NSE4	Isoleucine--tRNA ligase, mitochondrial	IARS2	2	5	7.2	1.055	7.40
424	P61978	Heterogeneous nuclear ribonucleoprotein K	HNRNPK	8	22	53.6	1.055	27.56
425	Q53FV1	ORM1-like protein 2	ORMDL2	3	3	21.6	1.054	34.10
426	Q8TCT9	Minor histocompatibility antigen H13	HM13	4	4	15.8	1.054	26.33
427	P56537	Eukaryotic translation initiation factor 6	EIF6	3	4	26.9	1.053	3.03
428	O75400	Pre-mRNA-processing factor 40 homolog A	PRPF40A	6	11	13.3	1.053	28.35
429	P46459	Vesicle-fusing ATPase	NSF	6	12	21.6	1.053	15.86
430	Q7Z7H5	Transmembrane emp24 domain-containing protein 4	TMED4	3	3	14.0	1.049	6.49
431	P15924	Desmoplakin	DSP	3	40	16.7	1.047	68.03
432	P46776	60S ribosomal protein L27a	RPL27A	4	4	31.5	1.047	28.13
433	Q8WUM4	Programmed cell death 6-interacting protein	PDCD6IP	6	11	16.6	1.047	44.84

Supplementary Table 1: Continued.

No.	Protein ID	Protein name	Gene name	No. of proteins [a)]	Seq. Pep. [b)]	Seq. Cov. % [c)]	ErbB2-digi /ErbB2 [d)]	Var. % [e)]
434	Q6DD88	Atlastin-3	ATL3	4	9	28.1	1.046	24.41
435	P19784	Casein kinase II subunit alpha	CSNK2A2	4	2	6.3	1.045	20.64
436	P55072	Transitional endoplasmic reticulum ATPase	VCP	4	41	52.1	1.045	37.46
437	Q12797	Aspartyl/asparaginyl beta-hydroxylase	ASPH	15	20	31.9	1.044	26.39
438	P84090	Enhancer of rudimentary homolog	ERH	2	2	25.0	1.042	6.07
439	Q9NP72	Ras-related protein Rab-18	RAB18	5	7	38.3	1.041	29.44
440	P43487	Ran-specific GTPase-activating protein	RANBP1	8	6	43.3	1.040	62.32
441	O43592	Exportin-T	XPOT	4	8	10.2	1.039	124.90
442	Q12906	Interleukin enhancer-binding factor 3	ILF3	20	30	50.0	1.038	31.45
443	A5YKK6	CCR4-NOT transcription complex subunit 1	CNOT1	7	4	2.0	1.038	31.11
444	Q16531	DNA damage-binding protein 1	DDB1	16	23	24.9	1.038	28.09
445	Q12905	Interleukin enhancer-binding factor 2	ILF2	2	12	51.0	1.038	39.59
446	O75915	PRA1 family protein 3	ARL6IP5	4	3	14.4	1.038	9.63
447	P05388	60S acidic ribosomal protein P0	RPLP0	14	13	42.3	1.037	35.15
448	Q07021	Complement component 1 Q subcomponent-binding protein, mitochondrial	C1QBP	3	6	24.1	1.037	30.55
449	P68400	Casein kinase II subunit alpha	CSNK2A1	6	12	39.7	1.036	21.34
450	Q9BQE5	Apolipoprotein L2	APOL2	2	4	13.1	1.036	52.78
451	Q99729	Heterogeneous nuclear ribonucleoprotein A/B	HNRNPAB	7	7	32.1	1.036	29.36
452	Q96HY6	DDRGK domain-containing protein 1	DDRGK1	2	3	14.7	1.034	36.57
453	Q13445	Transmembrane emp24 domain-containing protein 1	TMED1	3	2	22.0	1.033	7.57
454	Q9Y5M8	Signal recognition particle receptor subunit beta	SRPRB	3	8	29.5	1.033	13.43
455	P02545	Prelamin-A/C	LMNA	8	29	46.5	1.032	28.38
456	O14497	AT-rich interactive domain-containing protein 1A	ARID1A	14	16	11.5	1.031	48.72
457	Q01081	Splicing factor U2AF 35 kDa subunit	U2AF1	12	5	22.1	1.031	66.60
458	P14625	Endoplasmin	HSP90B1	4	51	54.0	1.030	41.48
459	P22307	Non-specific lipid-transfer protein	SCP2	7	5	32.1	1.029	9.72
460	P83731	60S ribosomal protein L24	RPL24	3	3	24.8	1.028	11.70
461	P61019	Ras-related protein Rab-2A	RAB2A	8	11	63.2	1.028	15.50
462	Q6P1M0	Long-chain fatty acid transport protein 4	SLC27A4	2	3	5.9	1.027	9.69
463	O00194	Ras-related protein Rab-27B	RAB27B	2	11	46.8	1.027	18.83

Supplementary Table 1: Continued.

No.	Protein ID	Protein name	Gene name	No. of proteins a)	Seq. Pep. b)	Seq. Cov. % c)	ErbB2-digi /ErbB2 d)	Var. % e)
464	P33992	DNA replication licensing factor MCM5	MCM5	4	18	29.8	1.025	26.73
465	P54578	Ubiquitin carboxyl-terminal hydrolase 14	USP14	6	12	35.9	1.024	15.37
466	Q12996	Cleavage stimulation factor subunit 3	CSTF3	2	4	9.2	1.022	21.26
467	Q01105	Protein SET	SET	5	12	47.9	1.022	15.46
468	Q29RF7	Sister chromatid cohesion protein PDS5 homolog A	PDS5A	4	15	15.9	1.020	80.13
469	Q13838	Spliceosome RNA helicase DDX39B	DDX39B	18	15	38.1	1.019	22.73
470	Q9Y5B9	FACT complex subunit SPT16	SUPT16H	4	27	27.7	1.019	46.92
471	Q8N766	ER membrane protein complex subunit 1	EMC1	5	13	19.1	1.019	28.29
472	P46781	40S ribosomal protein S9	RPS9	5	16	58.8	1.019	14.63
473	Q13123	Protein Red	IK	5	2	9.4	1.019	35.00
474	P20340	Ras-related protein Rab-6A	RAB6A	13	11	53.4	1.019	16.00
475	P10316	HLA class I histocompatibility antigen, A-69 alpha chain	HLA-A	53	6	21.9	1.019	17.12
476	P38606	V-type proton ATPase catalytic subunit A	ATP6V1A	4	9	20.9	1.018	29.15
477	Q7Z406	Myosin-14	MYH14	8	46	28.7	1.018	39.96
478	Q96GC9	Vacuole membrane protein 1	VMP1	13	3	15.1	1.018	13.80
479	Q13641	Trophoblast glycoprotein	TPBG	1	4	12.9	1.017	28.93
480	O00268	Transcription initiation factor TFIID subunit 4	TAF4	2	2	13.0	1.016	24.98
481	Q53GS9	U4/U6.U5 tri-snRNP-associated protein 2	USP39	5	7	19.4	1.015	25.49
482	O75937	DnaJ homolog subfamily C member 8	DNAJC8	2	5	33.2	1.015	16.22
483	P62244	40S ribosomal protein S15a	RPS15A	7	8	60.8	1.015	25.29
484	P62316	Small nuclear ribonucleoprotein Sm D2	SNRPD2	4	7	44.9	1.014	12.74
485	P23246	Splicing factor, proline- and glutamine-rich	SFPQ	4	22	39.3	1.013	56.63
486	P08238	Heat shock protein HSP 90-beta	HSP90AB1	4	50	54.1	1.013	31.02
487	Q92922	SWI/SNF complex subunit SMARCC1	SMARCC1	2	11	13.0	1.013	26.20
488	Q9H3N1	Thioredoxin-related transmembrane protein 1	TMX1	2	6	18.9	1.013	46.74
489	O95470	Sphingosine-1-phosphate lyase 1	SGPL1	3	6	13.6	1.013	39.70
490	Q9HAV4	Exportin-5	XPO5	3	6	6.1	1.012	25.62
491	Q96P11	Putative methyltransferase NSUN5	NSUN5	7	2	6.6	1.011	68.03
492	Q12981	Vesicle transport protein SEC20	BNIP1	4	2	9.2	1.010	14.78
493	P67870	Casein kinase II subunit beta	CSNK2B	3	3	15.8	1.008	24.61
494	Q96FV9	THO complex subunit 1	THOC1	6	5	10.8	1.007	8.24

Supplementary Table 1: Continued.

No.	Protein ID	Protein name	Gene name	No. of proteins a)	Seq. Pep. b)	Seq. Cov. % c)	ErbB2-digi /ErbB2 d)	Var. % e)
495	Q14683	Structural maintenance of chromosomes protein 1A	SMC1A	5	35	30.8	1.006	63.72
496	P49736	DNA replication licensing factor MCM2	MCM2	6	28	41.5	1.006	30.43
497	O95197	Reticulon-3	RTN3	5	2	5.5	1.005	14.40
498	P62304	Small nuclear ribonucleoprotein E	SNRPE	2	3	29.3	1.005	2.68
499	Q96FW1	Ubiquitin thioesterase OTUB1	OTUB1	7	5	19.6	1.005	27.30
500	P27449	V-type proton ATPase 16 kDa proteolipid subunit	ATP6V0C	1	2	31.6	1.004	1.53
501	P07900	Heat shock protein HSP 90-alpha	HSP90AA1	7	48	49.6	1.004	47.25
502	Q15005	Signal peptidase complex subunit 2	SPCS2	5	6	38.2	1.003	41.25
503	Q9Y5Q9	General transcription factor 3C polypeptide 3	GTF3C3	5	4	6.0	1.003	5.61
504	Q9Y265	RuvB-like 1	RUVBL1	6	9	27.0	1.003	38.91
505	Q15773	Myeloid leukemia factor 2	MLF2	4	2	15.7	1.001	34.57

a) Indicates the number of proteins identified by MS.

b) Indicates the number of peptides identified per protein by MS.

c) The percent coverage of identified peptides out of the full protein.

d) The ratio of ErbB2-digi/ErbB2; represents the quanitification of proteins in MCF7-p95ErbB2 cells treated with digitonin compared with untreated MCF7-p95ErbB2 cells. The experiment was performed twice with comparable results.

e) Variability gives the relative deviation of quantified peptide ratios from one protein.
